# Quantum-inspired modeling of distributed intelligence systems with artificial intelligent agents self-organization

**DOI:** 10.1038/s41598-024-65684-z

**Published:** 2024-07-04

**Authors:** A. P. Alodjants, D. V. Tsarev, A. E. Avdyushina, A. Yu Khrennikov, A. V. Boukhanovsky

**Affiliations:** 1 ITMO University, St. Petersburg, Russia 197101; 2 International Center for Mathematical Modeling in Physics, Engineering, Economics, and Cognitive Science Linnaeus University, 35195 Vaxjo-Kalmar, Sweden

**Keywords:** Quantum information, Information theory and computation

## Abstract

Distributed intelligence systems (DIS) containing natural and artificial intelligence agents (NIA and AIA) for decision making (DM) belong to promising interdisciplinary studies aimed at digitalization of routine processes in industry, economy, management, and everyday life. In this work, we suggest a novel quantum-inspired approach to investigate the crucial features of DIS consisting of NIAs (users) and AIAs (digital assistants, or avatars). We suppose that *N* users and their avatars are located in *N* nodes of a complex avatar - avatar network. The avatars can receive information from and transmit it to each other within this network, while the users obtain information from the outside. The users are associated with their digital assistants and cannot communicate with each other directly. Depending on the meaningfulness/uselessness of the information presented by avatars, users show their attitude making emotional binary “like”/“dislike” responses. To characterize NIA cognitive abilities in a simple DM process, we propose a mapping procedure for the Russell’s valence-arousal circumplex model onto an effective quantum-like two-level system. The DIS aims to maximize the average satisfaction of users via AIAs long-term adaptation to their users. In this regard, we examine the opinion formation and social impact as a result of the collective emotional state evolution occurring in the DIS. We show that generalized cooperativity parameters $G_i$, $i=1,\dots,N$ introduced in this work play a significant role in DIS features reflecting the users activity in possible cooperation and responses to their avatar suggestions. These parameters reveal how frequently AIAs and NIAs communicate with each other accounting the cognitive abilities of NIAs and information losses within the network. We demonstrate that conditions for opinion formation and social impact in the DIS are relevant to the second-order non-equilibrium phase transition. The transition establishes a non-vanishing average information field inherent to information diffusion and long-term avatar adaptation to their users. It occurs above the phase transition threshold, i.e. at $G_i>1$, which implies small (residual) social polarization of the NIAs community. Below the threshold, at weak AIA–NIA coupling ($G_i\le 1$), many uncertainties in the DIS inhibit opinion formation and social impact for the DM agents due to the information diffusion suppression; the AIAs self-organization within the avatar–avatar network is elucidated in this limit. To increase the users’ impact, we suggest an adaptive approach by establishing a network-dependent coupling rate with their digital assistants. In this case, the mechanism of AIA control helps resolve the DM process in the presence of some uncertainties resulting from the variety of users’ preferences. Our findings open new perspectives in different areas where AIAs become effective teammates for humans to solve common routine problems in network organizations.

## Introduction

### Background

Current communication technologies, such as the Internet, provide great opportunities for information exchange and cooperation. The interaction between people makes an essential social impact in networks and occurs due to communication tools (messages, tweets, texts, etc.) despite physical distances, national, cultural, or other differences. It is worth highlighting the dissemination of knowledge and expertise that can be effectively realized in various distributed networks by communities of human experts^1,2^. Contemporary networks establish so-called distributed intelligence systems (DISs) as a multidisciplinary paradigm for current society organization^3^. The rapid development of artificial intelligence systems has made them a critical part of decision-making (DM) and information exchange in networks. DISs may generally be recognized as multiagent systems possessing natural (humans) and artificial intelligent agents (AIAs), which are represented as digital assistants, or “avatars”. As a rule, the purposes of various DISs presume different strategies for the interaction of AIAs with each other and with natural intelligence agents (NIAs) within some complex networks; these strategies may become a source of uncertainties, risks, and complexity in the DIS, e.g.^4^. The capacity and complexity of AIA–NIA interaction and cooperation currently represent a challenging imperative, for example, for the Internet of Things^5^.

AIAs can perform various (dynamic) functions as a part of teams as well as can work independently on individual tasks due to advances in artificial intelligence^6^. By interacting with NIAs in social or some specialized networks, AIAs can not only be evaluated as advanced tools for performing routine tasks but more often as teammates for solving problems together with humans^7^. As a result, AIAs can interact with each other, which promotes the formation of AIAs’ “social groups” with their own opinions and inherent preferences about the problems and statements discussed in a network. In turn, humans facing artificial intelligence agents produce natural reactions, that are emotionally colored responses of the NIA to AIA’s actions and suggestions. Such responses may be formally accounted through “like”/“dislike” choices (decisions) delivered to AIAs within short response times. In this regard, it is unclear how collective artificial intelligence self-organization might be useful for NIA target communities to achieve their goals, and whether it is controllable or not. Another question is how AIAs opinions and offers can change the cognitive (emotional) states of NIAs target communities through interaction with them for a long enough time. In this work, we pursue answers applying quantum theory formalism to DM agents, which promise to become useful tools for accurate description of psychological and social features^8,9^.

### Related works

Before proceeding to the main part of this article, the core of the work is worth explaining in the context of relevant studies. Recently in^10^, we experimentally studied a DIS that consists of pairs of students (users) and their digital assistants (avatars) connected by an “avatar–avatar” graph, aimed to help students reduce some routine work in the framework of online regime education. The DIS had the goal to maximize the average satisfaction of users as a result of AIA–NIA interaction. It was shown that the DIS exhibits a complex behavior within short time slots even for simple interaction rules established initially between NIAs and AIAs. The results demonstrated that the dynamics of the DIS are complicated because of the temporal interplay between the agents’ probabilistic behavior and network topology, which was considered static. Moreover, the problem of how to evaluate the satisfaction of users over a long time arises. More generally, we can speak here about the long-term adaptivity or viability of digital assistants in multiagent systems, cf.^11^.

To elucidate the open problems established in this article, we suggest a novel quantum-inspired (Hamiltonian) approach for opinion formation in a multiagent (AIA–NIA ) coupled system, cf.^12^. To specify the elementary (“microscopic”) processes occurring in the DIS, we refer to quantum probability theory (QPT) and the Hilbert space to describe both AIAs and NIAs states, which characterize the agents’ probabilistic choice under uncertainty. In the last decades, the QPT approach to statistical problems has become prominent for explaining human cognitive behavior in some specific DM problems^8,13–16^. The QPT approach is also useful for human–AI specification problems, cf.^17,18^. The approach looks suitable to characterize the open systems that possess many various (apriori unknown) sources of uncertainties, cf.^9,19^. The quantum-inspired approach we suggest presumes to introduce an appropriate Hamiltonian connected to the objective function for the DIS under discussion, cf.^10^. In particular, the solution of Heisenberg-Langevin equations obtained from the Hamiltonian may be recognized as a result of some optimization procedure performed for a mathematically NP-hard problem that enables to description of the DIS long-term behavior. Recently, the Hamiltonian formalism has been successfully used in the development of heuristic (quantum-inspired) algorithms for solving NP-hard optimization problems, where specific (Ising-like) quantum Hamiltonians are mapped onto relevant objective functions to be minimized^20^. In this regard, our approach can be also recognized as quantum-inspired. Notably, in this work we do not examine the speedup problem actual for large-scale optimization; this problem is too important and should be studied separately, cf.^21^. However, the quantum physical approach we use represents a direct path for quantum and/or quantum-like simulators implementation for multiagent system optimization^22,23^.

In this work, we examine opinion formation and social impact as a result of collective emotional state evolution occurring in the DIS. In the framework of Russell’s circumplex model of emotions, we characterize NIAs emotional states with the help of arousal and valence variables which represent discrete quantum variables in our case. In^24^, we have shown that equilibrium phase transitions occurring in the social network system can also describe a significant change in the collective emotional state of some target social communities. In this study, we consider an open (driven-dissipative) environment of DIS that exhibits non-equilibrium phase transition (cf.^25^) and possesses so-called, social laser (solaser) features that we examined in^26,27^. Our approach is based on the paradigm of s- (socially actual information) field formation, which can be enhanced due to network peculiarities. This feature is the core of important differences between our approach and currently used opinion formation models based on Ising-like Hamiltonian where the information field itself is “tracing out”, cf.^28,29^. In particular, in^27^ we showed that simple rules borrowed from quantum physics and applied to DM agents at the microscopic level of consideration provide the accurate characterization of DM agents community. At the macroscopic level of a social system, these rules result in cascades and information diffusion, which play a fundamental role in current social systems. Practically, diffusion processes are important for the dissemination of knowledge, ideas, and innovations in the economy, finances, and other areas of human life and activity, cf.^30–32^. This work aims to clarify the conditions for the long-term diffusion establishment in the DIS, as a clear benchmark of long-term avatars adaptability to their users.

Noteworthy, for the model described in^27^, DM agents have an opportunity for tight cooperation due to their interaction, which is implemented through coupling with common information fields inherent to a given network community. By analogy with quantum physics (see e.g.^33^), in^34^ we introduced a new (network enforced) cooperativity parameter that accounts for an open driven-dissipative character of information-assisted dynamical processes occurring in the DIS. We demonstrated that the introduced parameter may be essentially enhanced for scale-free networks. Growing frequent communications between DM agents via coupling with information fields that enhance the cooperativity parameter reflects the growing activity of DM agents within the specific network. Such an activity may be recognized as an improvement of cooperation between agents leading to the establishment of socially homogeneous groups, as considered in the framework of game theory, see e.g.^35,36^. Notably, in^37^ authors arrive at the same conclusion examining the Prisoner’s Dilemma and the Snowdrift game in the framework of the evolutionary games approach performed on a scale-free network. In particular, in^37^ it is demonstrated that cooperation becomes a dominating feature for the entire range of parameters in both games and essentially depends on network peculiarities, cf.^38–40^. Here, we examine more general, power-law degree distribution $p(k)\propto k^{-\eta }$ graphs for modeling AIA–AIA networks; $\eta$ is the degree exponent; *k* is node degree continuous variable [see Eq. (25)]. The main properties of this network relate to the formation of hubs (see e.g.^41^), which, as we show below, determine the cooperative properties of NIAs and specify self-organization effects for AIAs.

The article is arranged as follows. In the “Background” and “Related works” sections we state the research motivation and significance and review relevant literature for the problems under discussion, respectively. In the “Quantum-inspired model of DIS” section we introduce the model of DIS to be examined in this work. Special attention is devoted to the specification of NIAs emotional states in the presence of information exchange with AIAs. In the “NIAs decision-making evaluation” section we discuss the possible features of the population imbalance variable that characterizes the pattern of the NIAs community collective decision within the DIS. The “Stationary states of DIS” section establishes one of the important approaches based on a steady-state solution for NIA population imbalance. This approach allows for studying NIAs self-organization processes within the network for certain specified NIAs decisions. In the “Network-free AIA–NIA interaction” section we represent the quantum-inspired model of AIA–NIA interaction without any network environment at all. We specify the cooperative parameter that we then explore for NIA–NIA cooperation characterization. In “DIS features in the presence of avatar–avatar network” sections and “Phase transition in DIS with AIAs self-organization” accurately determine the DIS properties that depend on NIA–NIA cooperation parameter features. We find it important to consider three limits when the cooperativity parameter is infinite, large enough, and small, respectively. Noteworthy, the parameter that we introduce in this work differs from the one discussed in the game theory, cf.^42^. In fact, the cooperativity parameter introduced in this work reflects potential cooperation abilities between DM agents that might be obtained from their activity level in the DIS. In “Opinion formation in the presence of weak AIA–NIA coupling” section describes the limit when such an activity is low enough when the coupling between AIA–NIA is weak. In “Opinions formation in the presence of AI agents adaptive control” section establishes important results on adaptive control of AIAs in the presence of weak coupling between users and their avatars. We suggest various scenarios for increasing the AIAs controllability in this case. The “Information diffusion in DIS” section specifies the opinion formation dynamically, i.e. beyond steady-state solutions. We pay attention to the information diffusion occurrence in the DIS that can be used as a benchmark of avatars long-term adaptivity to their users. Some specific theoretical, numerical, and computational methods that we use in this work are discussed in the “DIS network model”, “Quantum-inspired model of DM agents”, and “Mean-field equations” sections, respectively. In “Discussion” we summarize the results obtained and present a vision for further research development.

**Figure 1:**
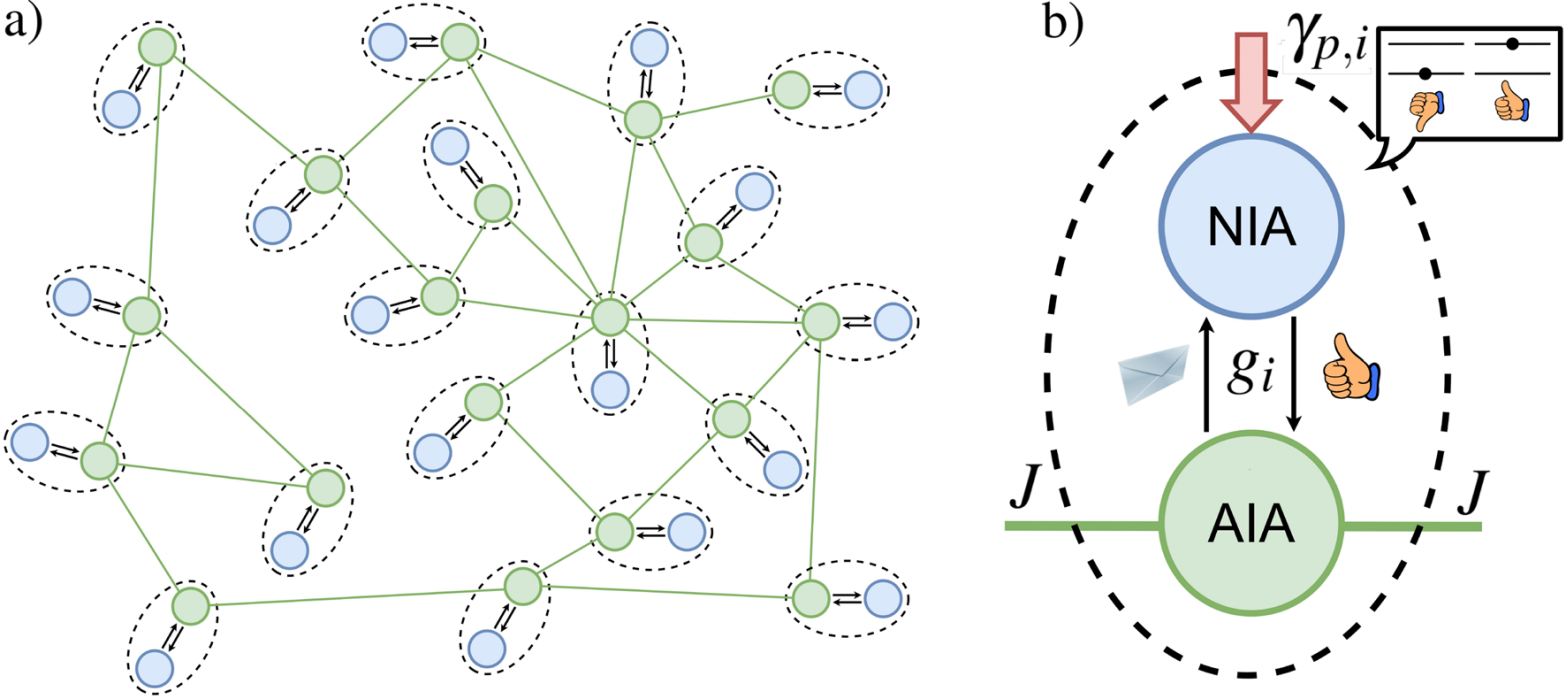
(**a**) Sketch of the distributed intelligence system (DIS) model that consists of AIA–NIA pairs located at each of *N* nodes; *J* is the avatar–avatar coupling strength. (**b**) Sketch of a single AIA–NIA pair, where avatars recommend information to the users received from the avatar–avatar network, assuming it to be relevant and useful for their users; users can accept or reject this information. $g_i$ is specific AIA–NIA coupling parameter that indicates frequency of *i*-th avatar–user communication; $\gamma _{p,i}$ is the information pumping rate. Other details are given in the text.

## Results

### Quantum-inspired model of DIS

Fig. 1 illustrates the DIS model we consider in this work; it implies the existence of two types of agents which are NIAs (users) and their digital assistants (avatars), AIAs, cf.^10^. We suppose that AIAs and their users (NIA) are located in the nodes of a complex avatar–avatar network, see Fig. 1a. Avatars can receive and transmit information to each other within this network, while the users are only associated with their digital assistants and cannot communicate with each other directly.

**Figure 2:**
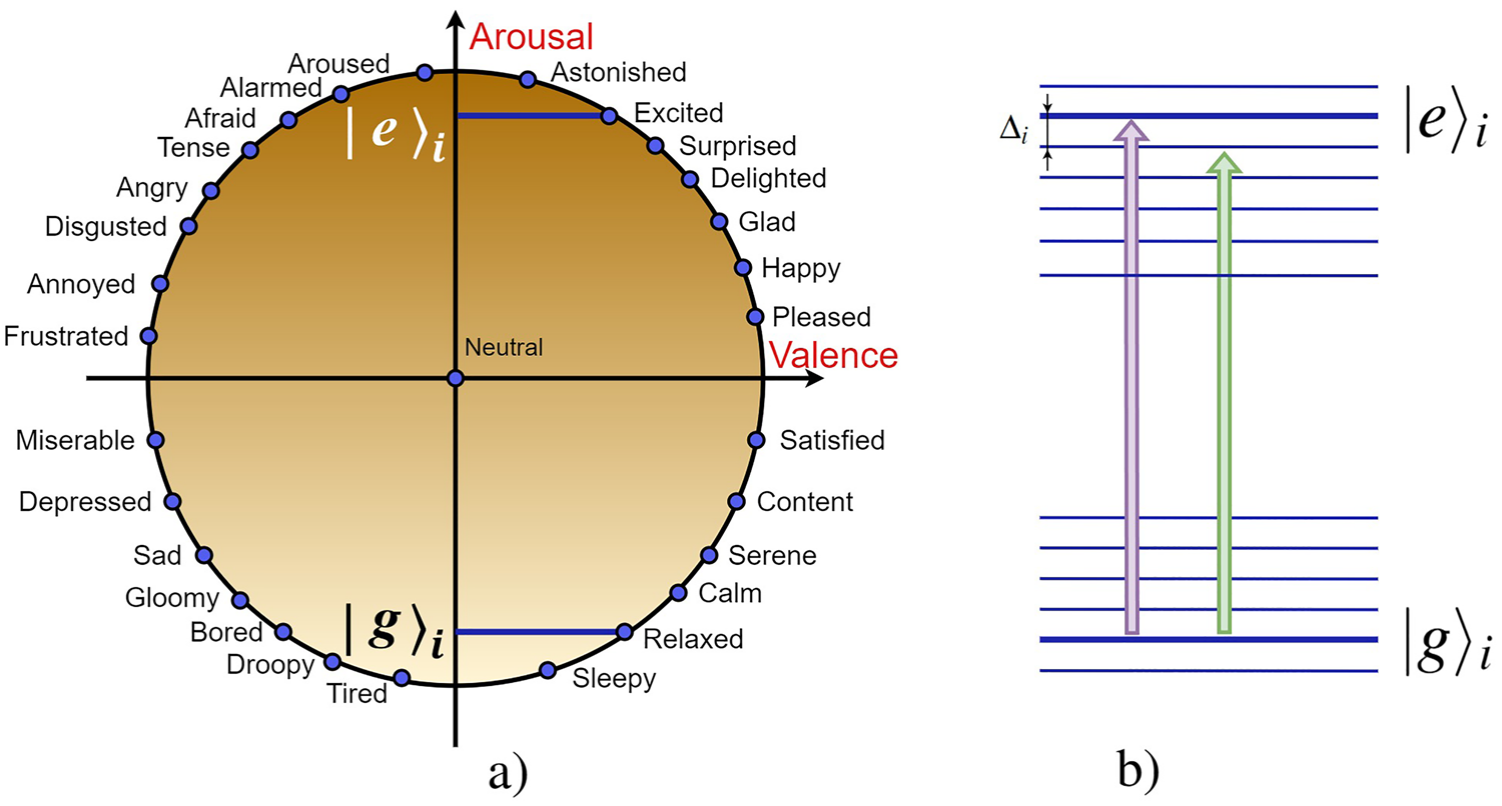
Mapping of (**a**) Russell’s circumplex model of affect onto (**b**) quantum-like two-level system (TLS) for the *i*-th NIA that interacts with information s-field; $i=1,2,\ldots , N$. Each of the thirteen emotional states of the right half of the circle in (**a**) are mapped to social energy levels of an effectively two-level system in (**b**). These levels are grouped around two mutually exclusive mental states $|g\rangle _i$ and $|e\rangle _i$, respectively (the bold lines). Two vertical thick arrows establish the changes in NIA mental state when they absorb s-photon; $\Delta _i$ is the detuning from the resonant transition.

Different individuals’ opinions are established through their interaction with their avatars and information that they can obtain from the outside of a network community with rate $\gamma _{p,i}$. In other words, users cannot discuss with each other some decisions directly. However, they can announce and read public messages (posts, for example) in the network and come to some mental state that we specify in the framework of the circumplex model of affect, e.g.^43^. The interaction of an avatar with its user is organized as follows. Users can accept, reject, or ignore this information; they show their attitude to the information presented by avatars making “like”/ “dislike” responses if the information is meaningful/useless for them, respectively, see Fig. 1b. The avatars’ opinions and preferences form through the avatar–avatar interaction in the network. The suggestions that avatars serve for the users reflect the average “collective mind” inherent to AIAs. Thus, avatars are self-organized within the complex network through information exchange and avatar–avatar interaction facilities. Avatars can disseminate information within the network and communicate with each other and with users simultaneously. The avatars’ goal is to maximize the users’ satisfaction within the DIS and keep them emotionally happy. Below, we elucidate the conditions when it occurs.

We propose a quantum-inspired approach for agent-based modeling of the DIS. In particular, each of the two DM states in our model we also recognize as mental states that relate to certain emotions of NIAs. Typically, basic human emotions are described by discrete categories^44^. The pairs of opposite primary emotions (acceptance–disgust, joy–sadness, anger–fear, anticipation–surprise) can represent some analog of “spin up” and “spin down” variables, respectively, e.g.^45,46^. To recognize the mental state of the *i*-th NIA ($i = 1,\ldots ,N$), we exploit the circumplex model of valence and arousal that indicates human emotions and their intensity, respectively, e.g.^43,47^, see Fig. 2a. Although the Russell model that exploits 2D plane (phase-space) visualization of basic emotions is not unique in cognitive sciences and has been debated for a long time (see, e.g. ^48^), it permits a relatively simple mathematical formalization and is inherent to current studies and technological applications within the human–computer interaction paradigm, e.g.^49^. Alternatively, it is possible to represent emotional (spin-like) states in the Bloch sphere that may be useful for using the Plutchik model of emotions, for example, c.f.^50^. However, as seen in Fig. 2b, the Russell model is more suitable for our case since it presumes a simple visualization of DM agents interaction with various information fields, which can change the emotional states of users. This is because the purpose of our analysis in this work is both the behavior of the two-level (cognitive) systems and the information field interacting with them.

We regard NIAs as so-called social atoms of the DIS^51^, see Fig. 2b. We choose two basic mental states $|g\rangle _i$ and $|e\rangle _i$ from the lower and upper halves of the circle, which provide negative and positive arousal levels, respectively, see Fig. 2a. We can also recognize states $|g\rangle _i$ and $|e\rangle _i$ as two mutually exclusive decisions (opinions or avatar suggestions) $\mathscr {S}_g$ and $\mathscr {S}_e$ in the DIS, respectively. Fig. 2b illustrates the resulting cognitive effectively two-level system (TLS) of NIA suitable to the description of information exchange in the DIS. Basic states $|g\rangle _i$ and $|e\rangle _i$ possess some social energies $E_{g,i}$ and $E_{e,i}$, which are reminiscent of the energies of a TLS ($E_{e,i} > E_{g,i}$) in quantum mechanics. The other emotional states with some social energies are shown in Fig. 2b by the blue horizontal lines according to their location on the circle in Fig. 2a. The light-violet arrow in Fig. 2b indicates the change of a user’s mental state from $|g\rangle _i$ to $|e\rangle _i$, when they obtain some significant information from the avatar. In other words, this information excites the user’s mental state. From Fig. 2, it is evident that the information obtained is thematically (contextually) “resonant” with transition frequency $\Omega _{0,i}=(E_{e,i}-E_{g,i})/\hbar$, i.e. energy $\hbar \Omega _{i}$ should be close to $\hbar \Omega _{0,i}$, as it takes place in quantum theory, e.g.^52^. Contrary, the light-green arrow in Fig. 2 demonstrates a non-resonant absorption of an s-photon by the user; after obtaining the information from their avatar, the user becomes excited and surprised; $\Delta _i\equiv \Omega _i-\Omega _{0,i}$ characterizes specific detuning of the user’s mental energy from its resonant value $E_{e,i}$ in this case, see Fig. 2. Thus, we project the variety of emotional states of NIAs represented by the valence discrete variable in Fig. 2a onto the ordinate axis of the TLS, and it may be established by a variety of detunings $\Delta _i$. In this case, characteristic frequencies $\Omega _{0,i}$ and detunings $\Delta _i$ are sufficient for the characterization of NIAs’ mental states under the interaction with AIAs.

Noteworthy, we suppose that NIAs exhibit primary neutral and positive valence emotions on average; although for arbitrary social networks such an assumption is difficult to fulfill. People become aroused because of some unpleasant or even tragic events occurring in their lives and tend to emotionally discuss them in social networks. In online communities, it is possible to come across envy, hate, and other extremely negative emotions, e.g.^53^. However, for a large number of specialized DIS networks, negative (extreme) emotions that possess positive arousal and negative valence, fortunately, are not common. Thus, the DISs we consider may be associated with different marketplaces, office-oriented networks, variety of networks focused on users’ hobbies and education.

We describe the information that the *i*-th avatar provides to the *i*-th user by the bosonic creation, $\hat{a}_i^{\dag }$, and annihilation, $\hat{a}_i$, operators, respectively; *i* enumerates the number of nodes in the network, i.e. the number of avatar–user pairs, $i=1,\ldots ,N$. In particular, operators $\hat{a}_i^{\dag }$, $\hat{a}_i$ specify the elementary processes of s-photon absorption and creation by the *i*-th user. Thus, we represent the Hamiltonian of the DIS in the form

1 $$\begin{aligned} \begin{aligned} {\hat{H} = \hbar \sum _{i=1}^{N}{\Bigg [\frac{1}{2}\Omega _{0,i}\hat{\sigma }_i^z + \Omega _i\hat{a}_i^{\dag }\hat{a}_i+g_i\left(\hat{a}_i\hat{\sigma }_i^+ + \hat{a}_i^{\dag }\hat{\sigma }_i^-\right)\Bigg ]}} {-\frac{\hbar J}{2}\sum _{i,j=1}^{N}{A_{ij}\left(\hat{a}_i^{\dag }\hat{a}_j + \hat{a}_j^{\dag }\hat{a}_i\right)},} \end{aligned} \end{aligned}$$

where the first two terms establish the NIA and AIA ensembles separately; $\hat{\sigma }^{z}_i = \hat{n}_{e,i}-\hat{n}_{g,i}$ is the operator of DM inversion for the *i*-th NIA; $\hat{\sigma }^{-}_i = |g\rangle _{ii}\langle e|$ and $\hat{\sigma }^{+}_i = (\hat{\sigma }^{-}_i)^{\dagger }$ are the ladder operators relevant to the excitation (polarization) and de-excitation of the *i*-th NIA as a simple TLS, cf.^52^. In (1) $\hat{a}_i^{\dag }\hat{a}_i$ is the s-photon number operator thematically “colored” by frequency $\Omega _i$. The third term in (1) is relevant to the *i*-th user–avatar interaction characterized by strength $g_i$. For our problem, parameter $g_i$ specifies how frequently the user communicates (checks messages, gives responses, etc.) with their avatar responsible for providing information. The last term in (1) establishes the interaction (information exchange) between avatars within the network that we describe by adjacency matrix $A_{ij}$; $J>0$ is the strength of this interaction (hereafter we set Planck constant $\hbar =1$ for simplicity of notation).

The mean-field equations that may be obtained with (1) look like [see also (31)]

2a $$\begin{aligned} \dot{E}_i=(-i\Delta _i-\kappa )E_i-ig_iP_i+iJ\sum _{j=1}^{N}{A_{ij}E_j}; \end{aligned}$$

2b $$\begin{aligned} \dot{P}_i=-\Gamma _i P_i+ig_i\sigma _iE_i; \end{aligned}$$

2c $$\begin{aligned} \dot{\sigma }_i=(\sigma _{0,i}-\sigma _{i})(\gamma _{p,i}+\gamma _{e,i})+2ig_i(E_i^*P_i-E_iP_i^*), \end{aligned}$$

where we made the definition $\sigma _{0,i}=\frac{\gamma _{p,i}-\gamma _{e,i}}{\gamma _{p,i}+\gamma _{e,i}}$. In (2) $E_i=\langle \hat{a}_i\rangle e^{i\Omega _{0,i} t}$ is the complex amplitude of the average coherent information field established by the *i*-th avatar within the DIS; $P_i=\langle \hat{\sigma }^-_i \rangle e^{i\Omega _{0,i} t}$ characterizes the excitation of the *i*-th user, $\sigma _i =\langle \hat{\sigma }_i^z \rangle$ represents the mean value of inversion operator $\hat{\sigma }_i^z$ that reflects an average mental (emotional) relationship of the *i*-th user to the information represented by their avatar, cf.^24,27^. In (2) $\Gamma _i$ is the *i*-th user excitation decay rate; $\kappa$ characterizes the average rate of information losses in the DIS. In this work, we suppose that $\Gamma _i$ uniformly distributed within some domain $\Gamma _i\in [\Gamma _{min}; \Gamma _{max}]$; $\Gamma _{min}$ and $\Gamma _{max}$ are minimal and maximal values of $\Gamma _{i}$ accesible to a particular NIA community, respectively.

In (2), $\Delta _i = \Omega _i-\Omega _{0,i}$ is the detuning that characterizes the efficiency of interaction of the *i*-th NIA with the avatar. To be more specific, we assume that $\Delta _i$ is also uniformly distributed within some domain $\Delta _i\in [\Delta _{min}; \Delta _{max}]$ that specifies how users are susceptible to a piece of information provided by their digital assistants; $\Delta _{min}$ and $\Delta _{max}$ are minimal and maximal values of $\Delta _{i}$, respectively. Therefore, users are maximally susceptible to the s-photons obtained from their avatars in the limit of $\Delta _i\simeq 0$ illustrated by the violet arrow in Fig. 2b. The limit of $\Delta _i^2\gg g_i^2$ corresponds to the off-resonance NIA–AIA interaction. In this case, the excitation probability of NIA rapidly vanishes, and individuals’ motivation to change their ground mental state is low enough. Such a cognitive behavior of NIAs is reminiscent of features of a two-level atom that interacts with the quantized irradiation, cf.^52^. Below we restrict ourselves by the limit of $\Delta _i^2\ll g_i^2$ that allows elucidating the influence of the avatars distributed within the complex network on the collective opinion formation and dissemination.

### NIAs decision-making evaluation

The DM process performed by the *i*-th user in the DIS is characterized by the mean value of inversion operator $\sigma _i =\langle \hat{\sigma }_i^z \rangle$ in (2). According to the circumplex model employed in this work (see Fig. 2), $\sigma _i$ is defined within domain $-1\le \sigma _i\le 1$. The limiting value, $\sigma _i = -1$, indicates the user’s decision that leaves them at the ground mental state, $|g\rangle _i$, supporting emotionally passive decision $\mathscr {S}_g$; $\sigma _i = 1$ describes the user’s excited mental state, $|e\rangle _i$, that corresponds to decision $\mathscr {S}_e$; value $\sigma _i = 0$ leaves the user’s position (decision) uncertain on average. We can define the total population imbalance averaged over the DIS of all network nodes as

3 $$\begin{aligned} \begin{aligned} \bar{\sigma }=\frac{1}{N\langle k\rangle }\sum _{i=1}^{N} k_i \sigma _i. \end{aligned} \end{aligned}$$

Variable $\bar{\sigma }$ reflects the average level of the users’ emotions and beliefs in the DIS. Parameter $\bar{\sigma }$ plays a crucial role in our work and can be evaluated for the DIS by measuring the users’ positive and negative responses. Below, we focus on a domain $0<\bar{\sigma }\le 1$ relevant to the users’ positive arousal and characterizing primary voting for $\mathscr {S}_e$. In other words, $\bar{\sigma }$ measures the satisfiability degree of NIAs in the DIS on average; it may be achieved within $0<\bar{\sigma }\le 1$ domain. In this case, we can assume that the majority of users are satisfied. On the other hand, value $\bar{\sigma } = 0$ may be recognized as some neutral (or uncertain) mental state for the DIS users, see Fig. 2.

Noteworthy, in social studies parameter $\bar{\sigma }$ (or its simpler version $\bar{\sigma }=\frac{1}{N}\sum _i\sigma _i$) is often interpreted as “social polarization”, that accounts for two opposite opinions inherent to the network actors, e.g.^54–56^. The polarized state appears as a result of agents’ homophily and echo chamber effect and features bimodal distribution for agents’ opinions^57^. The maximal social polarization occurs at $\bar{\sigma }=0$, which accounts for an approximately equal number of actors that exhibit opposite opinions^58^. Such an interpretation contradicts the solaser approach that is based on the quantum physics definition presuming that the polarization of a TLS is relevant to $p_i$, see (31) and e.g.^59^. However, following social science terminology in this work, we can also regard variable $\bar{\sigma }$ as average social polarization, while connecting $p_i$ with the socially excited state of NIAs.

### Stationary states of DIS

First, it is necessary to study conditions for coupled AIA–NIA opinion formation at the steady-state. In particular, we consider stationary states for Eq. (2), which can elucidate a variety of characteristic opinions (modes) that occur in the DIS. Without any interaction (communication) between NIA and AIA we can set $g_i = 0$ in Eq. (2c), which implies a steady-state solution of $\sigma _i$ in the form

4 $$\begin{aligned} \begin{aligned} \sigma _{i}\simeq \sigma _{0,i}=\frac{\gamma _{p,i}-\gamma _{e,i}}{\gamma _{p,i}+\gamma _{e,i}}, \; \; \; i=1,\ldots ,N. \end{aligned} \end{aligned}$$

Eq. (4) characterizes the inherent preferences of the *i*-th NIA determined by pumping rate $\gamma _{p,i}$ and spontaneously occurring decisions with decay rate $\gamma _{e,i}$. Notably, if the external information pumping rate is high enough and $\gamma _{p,i}>\gamma _{e,i}$, the user becomes initially excited, i.e. $\sigma _{0,i}>0$ and is more likely to support decision $S_e$. Otherwise, for $\gamma _{p,i}<\gamma _{e,i}$, the users represent a passive environment and tend to support decision $S_g$. At $\gamma _{p,i}=\gamma _{e,i}$ the users have no preferences and $\sigma _{0,i}=0$. We can assume that in this case, on average users remain uncertain in their decision.

In the first order of perturbation theory, we can assume that population imbalance $\sigma _i$ is given by Eq. (4). Now for the given population imbalance, $\sigma _{0,i}$, we can monitor NIA’s excitation level and s-field behavior, which may be recognized as a limit of social impact occurring within the DIS and carried out by AIAs through the network, cf.^60^. Stationary solutions of Eq. (2) imply variables $P_i(t) = \mathscr {P}_i e^{-i\omega t}$ $E_i(t) = \mathscr {E}_i e^{ - i\omega t}$, which evolve in time with frequency $\omega _i$ that characterizes the evolution of the *i*-th NIA’s excitation and information field in the network, respectively. Substituting $P_i(t)$ and $E_i(t)$ into Eq. (2) [see also Eqs. (31), (32)], we obtain

5 $$\begin{aligned} \left\{ (\omega -\Delta _i + i\kappa )\left( \omega + i\Gamma _i\right) + \sigma _{0,i} g_i^2\right\} \mathscr {E}_i + J\left( \omega + i\Gamma _i\right) \sum _{j=1}^{N}{A_{ij}\mathscr {E}_j}=0. \end{aligned}$$

Equation (5) describes stationary s-field formation in the DIS, supported by avatar–avatar interaction that occurs at some population imbalance $\sigma _{0,i}$ and is relevant to some level of user excitation. The set of frequencies $\omega$ defined by (5) may be associated with the variety of opinions occurring in the DIS. Notably, the structure of (5) is similar to that of the De Groot-Friedkin model of opinion formation, cf.^61^. In particular, the first term in (5) characterizes the self-appraisals of individual avatars. The second term in (5) describes the cooperation of avatars; in practice, it is important to elucidate the influence of this term on the properties of the DIS in detail.

### Network-free AIA–NIA interaction

First, let us examine Eq. (5) in the limit of isolated AIA–NIA pairs, i.e. at $J = 0$. In this limit, each *i*-th node avatar–user pair possesses two opinion eigenfrequencies. The solution of Eq. (5) gives

6 $$\begin{aligned} \omega _{1,2}=\omega _{1,2,i} = \frac{1}{2}\Bigg ({\Delta _{i}-i\xi _{+,i} \pm \sqrt{(\Delta _{i} - i\xi _{-,i})^2-4\sigma _{0,i} g_i^2}}, \Bigg ), \end{aligned}$$

where $\xi _{\pm ,i}\equiv \kappa \pm \Gamma _i$ represent effective losses of information in the DIS. For the chosen *i*-th avatar–user pair, Eq. (6) characterize the eigenfrequencies of two linearly coupled oscillators. In general, $\omega _{1,i}$ and $\omega _{2,i}$ are complex numbers. The imaginary parts of $\omega _{1,2,i}$ describe the social field reinforcement or its attenuation that is inherent to the *i*-th avatar–user specific opinions in the DIS.

**Figure 3:**
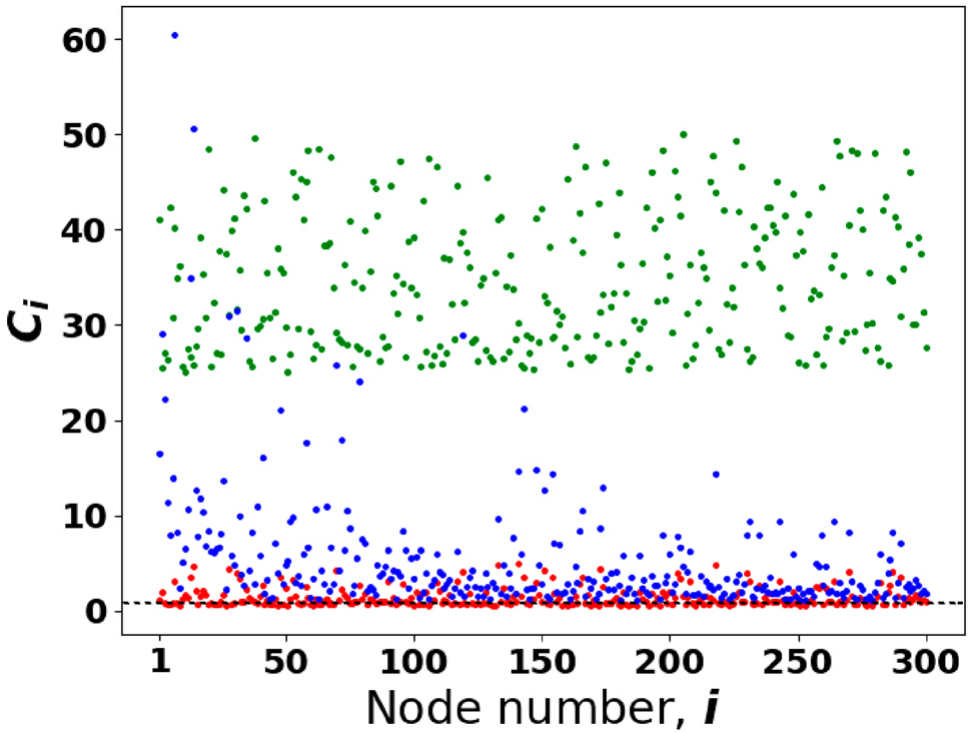
Distribution of NIAs cooperativity parameters $C_i$ for avatar–avatar complex network possessing constant user–avatar coupling strength $g_i=g=1$, $\kappa = 0.1$, $\Gamma _i\in [0.2; 0.4]$ (the green dots); $\kappa = 1$, $\Gamma _i\in [0.2;1.8]$ (the red dots), and node degree dependent strength $g_i=\sqrt{k_i}$ with $\kappa = 1$, $\Gamma _i\in [0.2;1.8]$ (the blue dots). The dashed line corresponds to $C_i = 1$; the number of nodes is $N = 300$.

We can recognize frequency difference

7 $$\begin{aligned} \Delta \omega _i\equiv \omega _{1,i}-\omega _{2,i}= \sqrt{(\Delta _{i} - i\xi _{-,i})^2-4\sigma _{0,i} g_i^2} \end{aligned}$$

as two oppositely directed and emotionally colored opinions that occur in the DIS due to avatar–user interaction, see (6). In quantum physics, $\Delta \omega$ defined in (7) characterizes the so-called Rabi splitting frequency that determines the frequency (energy) gap between two eigenfrequencies and appears due to the matter–field interaction^33^. In the case of the DIS, we can recognize $\Delta \omega$ as an emotionally enforced opinion gap that results from AIA–NIA communication. In other words, communication between the user and their avatar can shift the user’s emotional state, opinions, and beliefs.

As seen from (7), at $\sigma _{0,i}\ne 0$ this gap depends on the set of parameters that characterize the strength of AIA–NIA coupling and their peculiarities in information processing. In particular, for our approach it is fruitful to introduce dimensionless cooperativity parameter $C_{i}$, $i=1,\ldots ,N$ (cf.^33^)

8 $$\begin{aligned} \begin{aligned} C_{i}= \frac{g_i^2}{\Gamma _i\kappa }, \end{aligned} \end{aligned}$$

that represents the combination of key parameters inherent to AIAs and NIAs features in the DIS. $C_{i}$ in Eq. (8) characterizes the abilities of the *i*-th user ($i = 1,\ldots ,N$) to cooperate with other NIAs through coupling with common information field established in avatar-avatar network, cf.^34^. Since users cannot communicate with each other directly, their possible cooperation is specified by parameters $g_i$ relevant to NIA–AIA coupling rates.

As seen from definition (8), for $g_i = 0$ parameter $C_{i} = 0$ that implies the suppression of the cooperation between the NIAs performed through the coupling with AIAs. Weak cooperation corresponds to large information losses $\Gamma _i$ and $\kappa$ and implies the fulfillment of inequality $C_{i}\ll 1$. On the other hand, a high level of NIAs cooperation corresponds to the condition

9 $$\begin{aligned} \begin{aligned} C_{i}\gg 1 \end{aligned} \end{aligned}$$

that implies a strong coupling of NIA with AIA. In this limit, parameters in (9) obey condition $\Gamma _i, \kappa \ll g_i$. Moreover, the ideal, infinitely large cooperation between NIAs can appear for $\Gamma _i=\kappa =0$.

In Fig. 3, we establish the distribution of cooperativity parameters $C_i$, which present interest in this work. The blue dots indicate a transition from weak to strong cooperation regimes. In particular, NIAs cooperation may be strong enough for some nodes (hubs) due to the large values of parameter $g_i$, which may be caused by continual communication between the user and their digital assistant that indicates the network enhancing effect. However, before examining this effect in detail, let us start from the instructive limit of opinion formation when cooperation is ideal for all nodes, i.e. $C_{i}\rightarrow \infty$.

**Figure 4:**
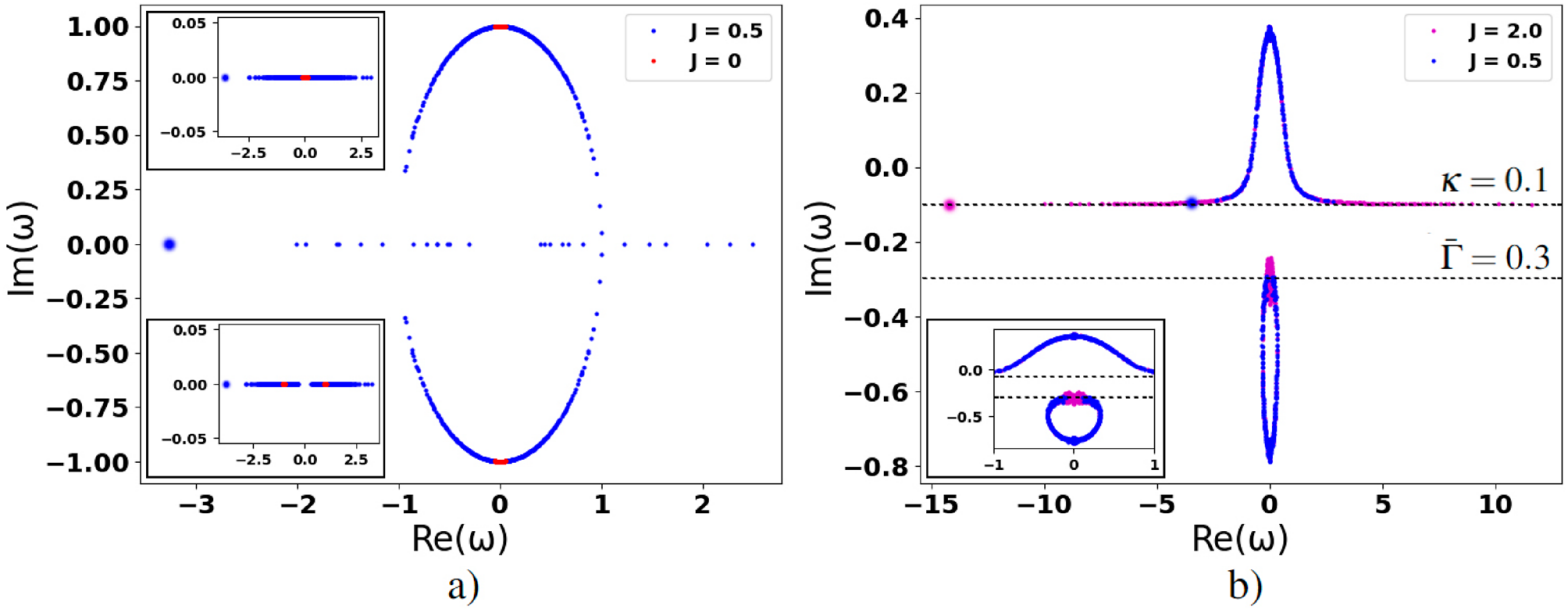
Dimensionless imaginary parts $\texttt {Im}(\omega )$ vs. the real ones, $\texttt {Re}(\omega )$, of the set of 2*N* eigenfrequencies $\omega$, which indicate opinion variety in the DIS for (**a**) $\Gamma _i = \kappa = 0$, $\sigma _{0,i}=1$; (**b**) $\Gamma _i\in [0.2;0.4]$, $\kappa =0.1$, $\sigma _{0,i}=0.3$. The other parameters are $N = 300$, $g_i = g = 1$, $\Delta _i\in [-0.1;0.1]$. Parameters $\Delta _i$ [and $\Gamma _i$ for (**b**)] are randomly and uniformly distributed variables. For the upper and lower insets in (**a**) $\sigma _{0,i}=0$ and $\sigma _{0,i}=-1$, respectively. The inset in (**b**) demonstrates the same dependence as the main plot (**b**) but within the window $-1\le \texttt {Re}(\omega )\le 1$. The bold blue and pink dots establish PEs. Algorithm S1 in Supplementary information explains the details.

In Fig. 4a we represent the dependencies of the eigenfrequencies imaginary parts vs. their real parts in the ideal case of $\Gamma _i=\kappa =0$. Here, we suppose $g_i=g=1$, $i=1,\ldots ,N$ for all AIA–NIA coupling rates. The frequency eigenvalues are situated symmetrically concerning positive and negative frequency detunings, see Fig. 4a. A real part of $\omega _i$ represents spectral characteristics that define a range of opinions in the DIS. The imaginary part of $\omega _i$ is relevant to discussions viability and their intensity. One can expect that the opinions with $\texttt {Im}(\omega _i)>0$ are reinforced while opinions, which pose $\texttt {Im}(\omega _i)<0$, are attenuated.

The red dots correspond to Eq. (6) for the network-free limit, $J = 0$, of isolated AIA–NIA states within DIS. Contrary, the blue dots in Fig. 4a establish the numerical solution of Eq. (5) and indicate opinion variety that occurs due to the avatar–avatar complex network given in Fig. 9. For Fig. 4a we suppose that $\sigma _{0,i}$ is represented by Eq. (4) and is the same for all AIAs. Practically, this assumption is applied to a homogeneous group of users, where each user apriori possesses emotions and beliefs that are close to some average level $\bar{\sigma }=\sigma _{i}\simeq \sigma _{0,i}$, which corresponds to average social polarization $\bar{\sigma }$, cf. (3), (4). This assumption may be also realized by choosing the appropriate information pumping rate $\gamma _{p,i}$ for different NIAs. The opinion formation in more general situations is considered in the next Section.

We take variable $\Delta _i$, which characterizes the variety of NIAs’ mental states in the DIS, as uniformly distributed within window $\Delta _i\in [-0.1; 0.1]$ relevant to the spread of the users’ initial opinions (and relevant emotions) in the DIS. In particular, for $\sigma _{0,i}=0$, all opinion eigenfrequencies are placed on the $\texttt {Im}(\omega _{1,2,i})=0$ line, see the inset to Fig. 4a. Practically, the blue dots show that all opinions occurring in the DIS are equivalent and widely represented in the DIS network. Noteworthy, the opinion gap vanishes, $\Delta \omega _i = |\Delta _{i}|$, for $\sigma _{0,i}=0$ that reflects insufficient external information obtained by users; they behave uncertainly (on average) in this limiting case.

The situation becomes more complicated when users are remarkably supported from the outside and $\sigma _{0,i}>0$. Eigenfrequencies $\omega _{1,i}$ and $\omega _{2,i}$ defined in (6) characterize the upper and lower branch opinions, respectively. At $\sigma _{0,i}=1$ opinion gap $\Delta \omega _i=\sqrt{\Delta _{i}^2 -4g_i^2}$ appears on the imaginary axis in Fig. 4a. Practically, such a feature manifests that not all opinions are equivalent in the DIS. For $\Delta _i^2\ll 1$, the opinion gap is purely imaginary $\Delta \omega _i=2i\sqrt{|\sigma _{0,i}|}g_i$. From Fig. 4a we can see that two oppositely oriented domains of red dots are located nearby $\texttt {Im}(\omega _{1,2,i})=\pm g_i=\pm g$, which is relevant to value $\sigma _{0,i}=1$ (in this work we use dimensionless parameter $g=1$). Practically, inequality $\texttt {Im}(\omega _{1,i})>0$ corresponds to the enhancement of the user’s opinion by the avatars due to strong AIA–NIA interaction. On the other hand, inequality $\texttt {Im}(\omega _{2,i})<0$ indicates opinions that are attenuated in time.

### DIS features in the presence of avatar–avatar network

Now, let us examine the role of avatar–avatar interaction in the $C_{i}\rightarrow \infty$ limit. Complex avatar–avatar network peculiarities possess a variety of opinions in the DIS at steady-state, which we establish in Fig. 4a by using the blue dots. For $J\ne 0$, we cannot associate eigenfrequencies with the nodes that possess arbitrary $k_i$. However, for the NIAs, which possess small $k_i$ and moderate *J*, we can assume $\mathscr {E}_j\simeq \mathscr {E}_i$ in (5). In this case, for characteristic frequencies $\omega _{1,2,i}$ one can obtain

10 $$\begin{aligned} \omega _{1,2,i} \simeq \frac{1}{2}\Bigg ({\delta _{i}-i\xi _{+,i} \pm \sqrt{(\delta _{i} - i\xi _{-,i})^2-4\sigma _{0,i} g_i^2}}\Bigg ), \end{aligned}$$

where $\delta _{i} = \Delta _i-\text {sign}(\mathscr {E}_i) J k_i$ is the new effective detunig that accounts DIS network peculiarities; $\text {sign}(\mathscr {E}_i)=\pm 1$ determines sign of the *i*-th eigenstate $\mathscr {E}_i$.

Equation (10) plays an important role in understanding opinion formation in the DIS. In particular, Eq. (10) manifests a hierarchy of opinions formed in the DIS and specified by $k_i$. In other words, the avatar–avatar network influences the mental state of each *i*-th user according to their avatar’s position within the network.

The contribution of avatar–avatar network to the opinion formation is easy to analyze analytically by setting $\xi _{\pm ,i}=0$ in (10); this limit corresponds to the blue dots in Fig. 4a. At $\sigma _{0,i}>0$ from Fig. 4a, it is evident, that three main domains manifest the variety of opinions in the DIS. Two domains are relevant to inequality $|\delta _i|<2g_i\sigma _{0,i}$ and may be obtained from (5) by (6), (10) in the form

11a $$\begin{aligned} \omega _{R,i}\equiv \texttt {Re}(\omega _{1,2,i})= \frac{1}{2}\delta _{i}; \end{aligned}$$

11b $$\begin{aligned} \omega _{I,i}\equiv \texttt {Im}(\omega _{1,2,i})=\pm \frac{1}{2} \sqrt{4 \sigma _{0,i} g_i^2-\delta _{i}^2}. \end{aligned}$$

From (11) and Fig. 4a, it follows that the dots, which demonstrate opinions variety in the DIS, are inhomogeneously distributed on the circle, $\omega _{R,i}^2 + \omega _{I,i}^2=\frac{1}{4}(4\sigma _{0,i} g_i^2-\delta _{i}^2)$. These dots characterize avatars who are strongly coupled with their users. The opinions (and emotional states) that possess frequency eigenvalues $\omega _{1,i}$ and belong to the upper half of the circle, $\omega _{I,i}>0$, are reinforced and disseminated by the avatars. The opinions that possess frequency eigenvalues $\omega _{2,i}$ ($\omega _{I,i}<0$) are discriminated in the DIS.

In general, the role of the avatar–avatar complex network in the DIS is established by the spectrum of the adjacency matrix, $A_{ij}$. The Perron-Frobenius theorem guarantees the existence of the non-degenerate positive maximum Perron eigenvalue (PE) (the bold dots for two various values of *J* in Fig. 4a) that corresponds to the eigenvector with all elements positive. Since $A_{ij}$ appears in Hamiltonian (1) with minus, the correspondent PE in Fig. 4a is negative. In particular, from Fig. 4a it is evident that complex avatar–avatar network peculiarities possess strong positions of the avatars placed on axis $\texttt {Im}(\omega _{1,2,i}) = 0$. The interaction effect of these avatars with their users looks not so strong due to large values of $|\delta _{i}|$ in comparison with effective parameter $2|\sigma _{0,i}|g\simeq 2g$. Moreover, in Fig. 4a we can the distinguish the dots, which belong to $\texttt {Re}(\omega _{1,i})>0$ and $\texttt {Re}(\omega _{2,i})<0$ domains, respectively. We can expect these dots to reflect opposite tendencies in opinions and emotions within the DIS. It is important to note that these states are possessed by avatars with high $k_i$.

In terms of the distributed intelligence systems, the PE corresponds to the most powerful avatar leader in the network. Noteworthy, the PE does not necessarily belong to a hub, i.e. the most connected node as it is for the scale-free network in Fig. 9a. Here, we use the eigenvector centrality criterion for $A_{ij}$ to obtain some principal analytical results using the PE. The eigenvector centrality characterizes the avatar node connection with the other high-rank nodes aiming to become more influential in the DIS. We establish the eigenvector centrality criterion in (28); $\mathscr {E}_{p,i}$ is the field eigenstate that corresponds to the positive PE, $\lambda _{p}$, cf.^62^. Substituting (28) into (5) for $\omega \equiv \omega ^{(p)}$, we obtain [cf. (10)]

12 $$\begin{aligned} \omega ^{(p)}_{1,2,i} = \frac{1}{2}\Bigg ({\delta _{p,i}-i\xi _{+} \pm \sqrt{(\delta _{p,i} - i\xi _{-})^2-4\sigma _{0,i} g_i^2}}\Bigg ), \end{aligned}$$

where $\delta _{p,i}=\Delta _i-J \lambda _{p}$ is the effective detunig of the *i*-th node that corresponds to the PE; $\xi _{\pm }\equiv \kappa \pm \bar{\Gamma }$; and $\bar{\Gamma }=(\Gamma _{max}+\Gamma _{min})/2$ is the average value of $\Gamma _i$ within domain $[\Gamma _{min}; \Gamma _{max}]$. The PE establishes a non-zero average s-field that we can introduce as

13 $$\begin{aligned} \begin{aligned} {\bar{\mathscr {E}}}_{p}=\frac{1}{N\langle k\rangle }\sum _{j=1}^{N} k_j \mathscr {E}_{p,j}=\frac{\lambda _{p} \mathscr {E}_{p,i}}{k_i}, \end{aligned} \end{aligned}$$

where $k_i=\sum _{j}A_{ij}$ is the *i*-th node degree. The last equality in (13) is written accounting (28) in the so-called annealed network approximation, $A_{ij}=\frac{k_ik_j}{N\langle k\rangle }$ that we examine below, cf.^27^. Then, substituting $A_{ij}$ into (5) and solving it for $\mathscr {E}_j$, we obtain

14 $$\begin{aligned} \begin{aligned} \mathscr {E}_{p,i}= - \frac{J(\omega ^{(p)}+i\bar{\Gamma })k_i {\bar{\mathscr {E}}}_{p}}{(\omega ^{(p)}-\Delta _i+i\kappa )(\omega ^{(p)}+i\bar{\Gamma })+ \sigma _{0,i} g_i^2}. \end{aligned} \end{aligned}$$

Eq. (14) characterizes the s-field at the *i*-th node that contains an AIA–NIA pair by the mean field induced by the avatar–avatar network. We can recognize Eq. (14) as the collective pressure, caused in the *i*-th node from the avatar network community. Combining (14) with (13), it is possible to obtain an estimation for the PE that is $\lambda _p\simeq \zeta$, see (27b).

### Phase transition in DIS with AIAs self-organization

Now let us analyze what happens in the DIS in the limit of finite cooperation between NIAs and AIAs, see (8). It is useful to consider two important limits for vital parameters $\Gamma _i$ and $\kappa$. As follows from (6), (7), for $\Gamma _i=\kappa$, $\xi _{-,i} = 0$ and opinion gap $\Delta \omega$ is independent on $\xi _{-,i}$ in this case. As a result, we obtain the same picture of opinion distribution in the DIS as in Fig. 4a, but shifted down from the $\texttt {Im}(\omega _i)$ axis on the value of $\xi _{+,i}$.

The picture significantly changes if $\Gamma _i\ne \kappa$ and $\xi _{-,i}\ne 0$. In Fig. 4b we represent the dependencies of imaginary parts of the eigenfrequencies vs. their real parts for the complex network established in Fig. 9. We also assume that parameter $\Gamma _i$, which determines non-equal abilities of NIAs, uniformly varies within domain $\Gamma _i\in [0.2;0.4]$, $i=1,\ldots ,N$. Strictly speaking, we analyze the case when cooperativity parameter $C_i>1$, see the green dots in Fig. 3, and cf. (8). For Fig. 4b, we assume that the users are supported by the information obtained from the outside, and $\sigma _{0,i}=0.3$.

Two groups of dots are closely located in the upper and lower parts of Fig. 4b and correspond to the main set of the eigenvalues of Eq. (5) and the eigenvalues of the adjacency matrix $A_{ij}$, cf.^63^. Their main feature is the ability to “attract” each other because the Eq. (5) matrix is non-Hermitian, cf.^64^. Such a non-Hermitian localization affects the spectral curves significantly. The dashed lines correspond to asymptotes with $\bar{\Gamma }=0.3$ and $\kappa =0.1$ and exhibit the opinion gap that separates these branches. In Fig. 4b, the opinion eigenfrequencies corresponding to these asymptotes are depicted by the nodes that possess large $k_i$.

In particular, the avatar–avatar coupling rate, *J*, essentially contributes to the total detuning, $\delta _{i}$, in (10). We can find the maximal value of the detuning for the node that corresponds to the PE. Assuming $|\delta _{p,i}-i\xi _{_,i}|^2\gg 4\sigma _{0,i} g_i^2$ in (12), characteristic frequencies can be obtained for the PE in the form

15a $$\begin{aligned} \omega ^{(p)}_{1,i}\simeq \delta _{p,i}- \frac{i}{2}(\xi _{-} +\xi _{+})=\delta _{p,i}-i\kappa ; \end{aligned}$$

15b $$\begin{aligned} \omega ^{(p)}_{2,i}\simeq \frac{i}{2}(\xi _{-} -\xi _{+})=-i\bar{\Gamma }. \end{aligned}$$

Eq. (15) well agree with the numerical results in Fig. 4b: values $\texttt {Im}(\omega ^{(p)}_{1,i})$ and $\texttt {Im}(\omega ^{(p)}_{2,i})$ approach the dashed lines denoting $\kappa =0.1$ and $\bar{\Gamma }=0.3$ in Fig. 4b.

It is important to note that Eq. (15) are independent on $g_i$. We can suppose that the nodes with large $k_i$, which are located along axis $\kappa =0.1$ in Fig. 4b, represent maximally influential AIAs in the avatar–avatar network. The connection with the users of relevant avatars is moderate.

In general, the Perron eigenvalues lie within $[\langle k\rangle , \, k_{max}]$, cf.^63^. The second PE for Eq. (2) is positive and located at the right-hand side of Fig. 4b. Thus, the self-organization of avatars with large $k_i$ within the network leads to their location on both sides of $\texttt {Re}(\omega _i)=0$; the influence of these avatars on each other is balanced. In practice, this means that avatars with high $k_i$ also form a specific agenda for discussions and then impose it to the users’ community. We can expect such avatars to be concerned with promoting and establishing “their ideas” in the network more than with satisfying the needs of their users. In some sense, the self-organization of influential avatars leads to two major opinion domains separated by the opinion gap formed by two dashed lines in Fig. 4b.

The situation significantly differs with the avatars that belong to the $\Lambda$-shape peak of the upper branch of opinions in Fig. 4b. The dots located in Fig. 4b in narrow (vertical) band $-g_i< \texttt {Re}(\omega )<g_i$ (at $g_i=g=1$) possess small $k_i$ and describe avatars that closely cooperate with their users, who possess definite opinions with small $\delta _i\simeq 0$. As a result, the avatars of these nodes are the first to pick up new ideas and innovations, which then begin to spread and reinforce themselves online. Their “mobility” is determined by the graph topology: these nodes with few connections are the least significant nodes of the opinion formation and located at the periphery of the network, see Fig. 9a.

The information field enhancement occurs at $\texttt {Im}(\omega )>0$. Thus, the condition for the transition from s-field attenuation regime to the amplification one, which occurs at $\texttt {Im}(\omega )=0$, represents a primary interest for investigating the influence and innovation spread in the network.

To find the frequencies of the dots in the vicinity of $\omega =\omega _i=0$, we can consider Eq. (10) assuming $\delta _i\simeq \Delta _i - J k_{min}$, where $k_{min}$ is the minimal value of the graph node degree. Characteristic frequencies in the vicinity of $\texttt {Re}(\omega _i)\simeq 0$ may be obtained in the form

16 $$\begin{aligned} \begin{aligned} \omega _{1,i}\simeq \frac{\delta _i}{2}, \quad \omega _{2,i}\simeq \frac{\delta _i}{2}-i\xi _{+,i}, \end{aligned} \end{aligned}$$

where we also assume that $\delta _i^2, |\delta _i|\Gamma _i \ll \xi _{+,i}^2$, and account condition

17 $$\begin{aligned} \begin{aligned} \sigma _{0,i} C_{i}=1. \end{aligned} \end{aligned}$$

Thus, as it follows from (16), frequency $\omega _{1,i}$ becomes real, $\texttt {Im}(\omega _{1,i})=0$, that manifests the transition to enhancement of social field, cf.^27^. The numerical calculations demonstrate that the s-field enhancement occurs when the dots in the upper part of Fig. 4b enter the real eigenvalues semi-plane, which is determined by condition (17).

Eq. (17) establishes the second-order non-equilibrium phase transition that occurs in the DIS and may be characterized by using *generalized* cooperative parameter

18 $$\begin{aligned} \begin{aligned} G_i\equiv \sigma _{0,i} C_{i}, \end{aligned} \end{aligned}$$

which takes into account not only the avatar–user coupling strength ($C_i\propto g_i^2$), but also apriori personal preferences of the users $\sigma _{0,i}$ in the presence of an external information pump. Condition (17) may also be recognized as a criterion for social laser transfer, which implies strong information field formation in the DIS, cf.^27^.

### Opinion formation in the presence of weak AIA–NIA coupling

**Figure 5:**
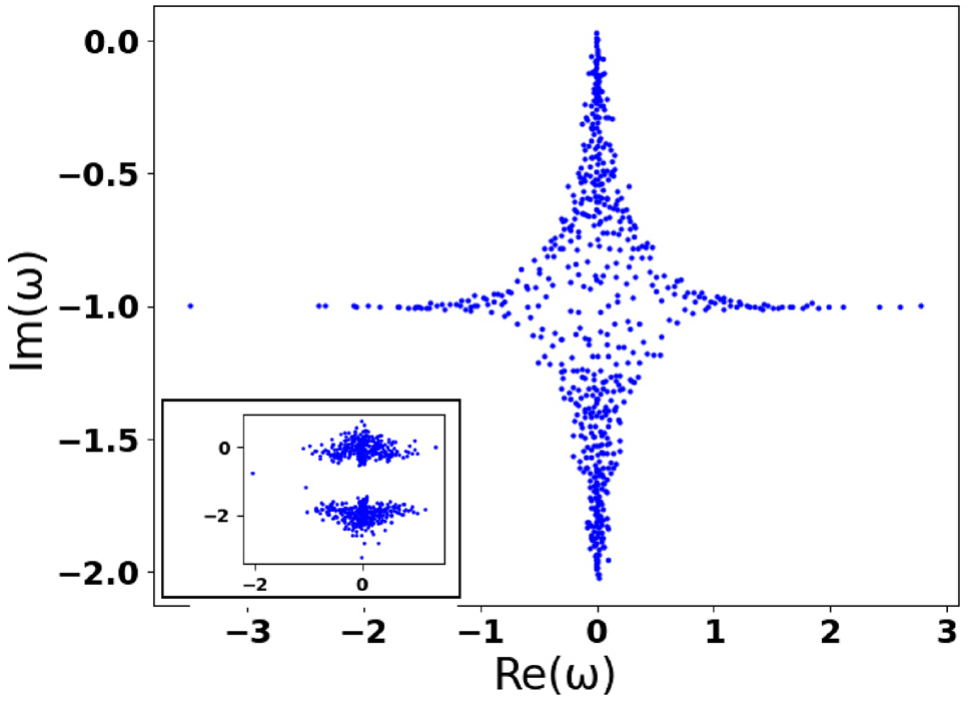
The same as in Fig. 4 but at $g_i = g = 1$, $\sigma _{0,i} = 0.3$, $\Gamma _i\in [0.2;1.8]$, $\kappa =1$. The inset demonstrates the same dependences for $g_i=\sqrt{k_i}$.

Now let us examine the remarkable case of weak coupling between AIAs and NIAs, which implies moderate values of $C_i\le 1$. This situation is practically relevant to large values of the large parameters $\kappa$ and $\Gamma _i$, respectively, see (8) and the red dots in Fig. 3. Strictly speaking, $C_i$ is randomly distributed in the vicinity of value $C_i=1$. Fig. 5 demonstrates uncertain features of the DIS in the limit of large $\Gamma _i$ and $\kappa$. At $\sigma _i=0.3$, generalized cooperation parameter $G_i\le 1$. The uncertainty comes from the users’ cognitive states, which are characterized by large $\Gamma _i$; it is random and uniformly distributed within larger window $\Gamma _i=[0.2;1.8]$. Practically, such a situation may be relevant to an inhomogeneous group of users who significantly differ from each other, and cooperation abilities between them look questionable. Coupling with avatars and obtaining relevant information from them is not enough for the users to overcome many uncertainties in this case, $g_i^2 < \bar{\Gamma }\kappa$. From Fig. 5, we can see that for averages $\bar{\Gamma }=\kappa =1$ the nodes with high $k_i$ are located along value $\texttt {Im}(\omega )=-1$. At the same time, Fig. 5 demonstrates the suppression of the opinion gap; the avatars with low $k_i$ chaotically occupy the area within domain $-1<\texttt {Re}(\omega )<1$. Thus, relatively weak coupling of the avatars with their users results in the absence of any preferences or influence in the DIS.

### Opinions formation in the presence of AI agents adaptive control

One of the vital problems in the framework of the DIS basic features is how NIAs can influence the processes occurring in the avatar–avatar network. Avatars’ self-organization within the network can introduce some uncertainties in the presence of weak cooperation of the users, see Fig. 5. Intuitively, we can expect an improvement of the DIS abilities with an enhancement of the AIA–NIA coupling strength. Strictly speaking, we should improve parameter $|\sigma _{0,i}| g_i^2$ adaptively, see e.g. (10); this simply means diminishing uncertainties in users’ decisions due to strong coupling with the environment using high information pump rate $\gamma _{p,i}$, see (4). However, the maximal value of parameter $\sigma _{0,i}$ is limited by magnitude $|\sigma _{0,i}|=1$.

We suggest another way to enhance NIAs cooperation in the DIS. Namely, increasing parameter $g_i$ we suppose that

19 $$\begin{aligned} g_i=g\sqrt{k_i}. \end{aligned}$$

Equation (19) means the inhomogeneous (node-dependent) avatar–user coupling strength adapted to the DIS as we imposed it in the solaser model, cf.^27^. In particular, to achieve a high level of coupling with avatars that occupy nodes with large $k_i$, their users should enhance communication with the avatars at least $\sqrt{k_i}$ times to keep them as effective digital assistants. The *i*-th user cooperativity parameter, in this case, grows as $C_i\propto k_i$, see (8). The distribution of relevant cooperativity parameters is shown by the blue dots in Fig. 3. High-level cooperativity between users is achieved for the nodes relevant to the hubs or high-centrality nodes. The inset in Fig. 5 shows the depenence $\texttt {Im}(\omega )$ on $\texttt {Re}(\omega )$ for $g_i$ given by (19) at $g=1$. The comparison of the main plot in Fig. 5 with the inset clearly demonstrates the formation of well-separated domains of opinions for $g_i$ given by Eq. (19) at $g=1$.

**Figure 6:**
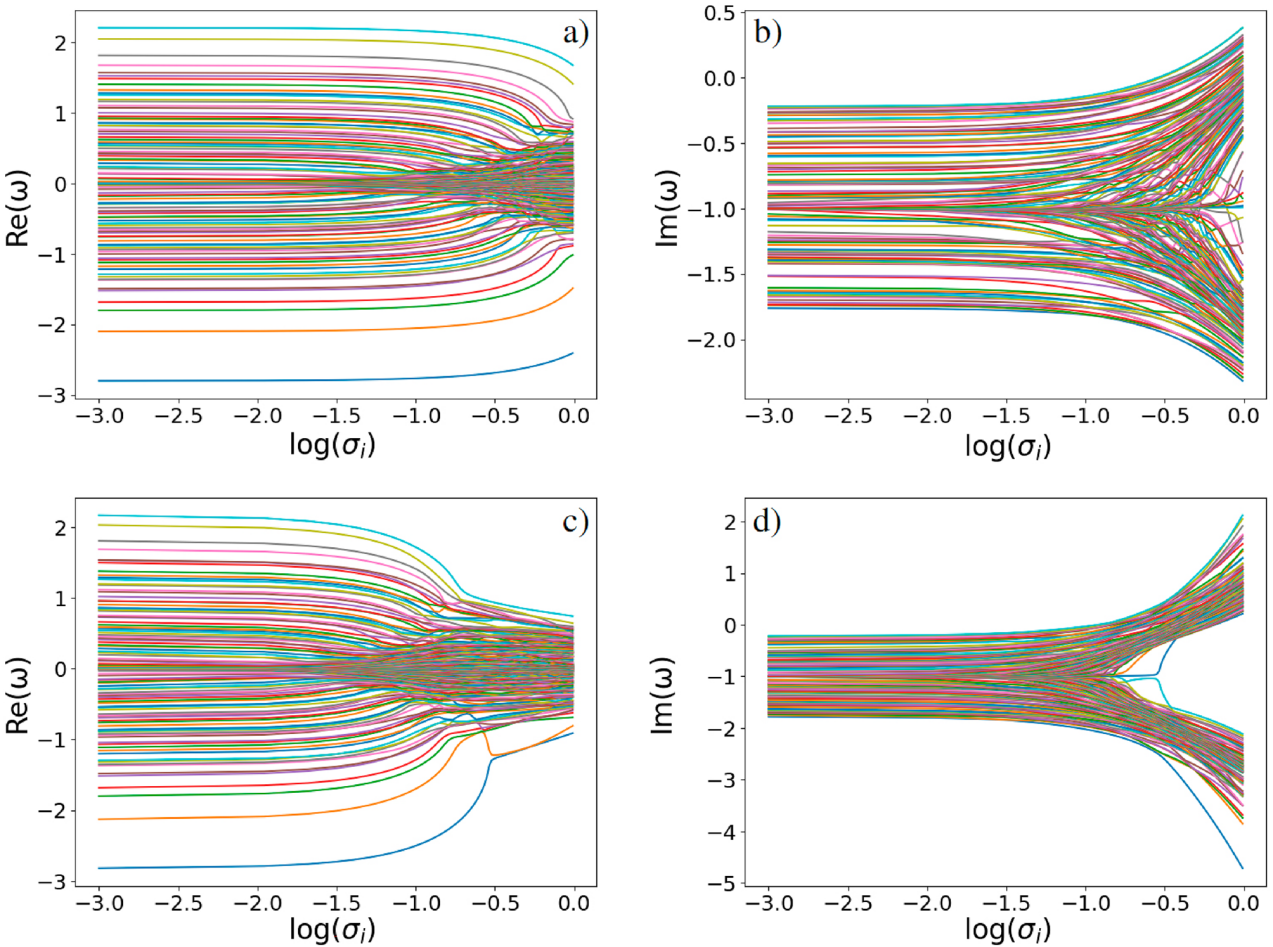
Dimensionless (**a**,**c**) real $\texttt {Re}(\omega )$ and (**b**,**d**) imaginary $\texttt {Im}(\omega )$ parts of eigenfrequencies $\omega$ for the DIS complex network vs. population inversion logarithm $\log (\sigma _i)$ within approach (3), (4) that implies $\bar{\sigma }=\sigma _{i}\simeq \sigma _{0,i}$. The avatar–user coupling strength is $g = 1$ for (**a**,**b**), and $g_i = g\sqrt{k_i}$ for (**c**,**d**). The other parameters are: $N = 100$, $\kappa =1$, $\Delta _i\in [-0.1;0.1]$ and $\Gamma _i\in [0.2;1.8]$ are random and uniformly distributed variables.

The results obtained here are summarized in Fig. 6; the figure demonstrates dimensionless dependence $\texttt {Re}(\omega )$ and $\texttt {Im}(\omega )$ as functions of population inversion logarithm $\log (\sigma _i)$ for the plots in Fig. 5. Here, we still adhere to the approximations presented in (3) and (4) that imply $\bar{\sigma }=\sigma _{i}\simeq \sigma _{0,i}$. In particular, Fig. 6a exhibits the key features of opinion range at $g = 1$ with increasing $\sigma _i$ from $\sigma _i = 0$ ($\log (\sigma _i)\rightarrow - \infty$) to $\sigma _i=1$ ($\log (\sigma _i)\rightarrow 0$ ), respectively. Each line in Fig. 6a corresponds to the dots located along the $\texttt {Re}(\omega )$ axis in Fig. 5. The lowest blue curve in Fig. 6a corresponds to the PE, $\texttt {Re}(\omega _{p,1})$, and changes slightly with the growth of $\sigma _i$. At the same time, the upper curves in Fig. 6a are relevant to the next PEs, which correspond to the rightmost dots in Fig. 5 and the powerful avatars in Fig. 9a. Thus, we can conclude that the most powerful avatars of the DIS tend to keep their position independent.

Contrary, as seen from Fig. 6a, the eigenfrequencies of the dots within area $-1<\texttt {Re}(\omega )<1$ (see Fig. 5) start “moving” towards each other when $\sigma _i$ grows, which demonstrates the opinion range narrowing. In other words, the avatars, which fall into domain $-1<\texttt {Re}(\omega )<1$, with their users tend to focus on suggestions supported by information pumping. However, the chaotization of each opinion trajectory seen in Fig. 6a occurs with increasing $\sigma _i$. It becomes more apparent if we analyze Fig. 6b. In particular, the total uncertainty and struggle of opinions are visible near value $\sigma _i = 1$.

The behavior of $\texttt {Re}(\omega )$ and $\texttt {Im}(\omega )$ significantly changes in the presence of adaptive control of AIAs for node-dependent coupling strength $g_i=\sqrt{k_i}$, see Fig. 6c,d. Fig. 6c shows that the opinions range in the DIS is essentially narrow due to opinion shaping management and node-dependent enhancement of avatar–user coupling parameters $g_i$. Notably, the avatars, which occupy nodes with high $k_i$, are also involved in this process, see the lower blue curve in Fig. 6c. The opinion gap that is evident from Fig. 6d manifests opinions separation. The behavior of the curves in Fig. 6d relevant to domain $\texttt {Im}(\omega )>0$ corresponds to the innovation spread and s-field amplification in the DIS. On the other hand, opinions within region $\texttt {Im}(\omega )<0$ are suppressed by the DIS.

### Information diffusion in DIS

Let us examine the information diffusion problem that occurs in the DIS due to AIA–NIA coupling within the network. We consider Eq. (2) in the limit of steady-state polarization ($\dot{P}_i=0$), which leads to

20a $$\begin{aligned} \dot{E}_i=(-i\Delta _i-\kappa )E_i+\kappa C_i\sigma _i E_i+iJ\sum _i{A_{ij}E_j}; \end{aligned}$$

20b $$\begin{aligned} \dot{\sigma }_i=(\sigma _{0,i}-\sigma _{i})(\gamma _{p,i}+\gamma _{e,i})-4 \kappa C_i \sigma _i|E_{i}|^2, \end{aligned}$$

Steady-state solution $\dot{\sigma }_i=0$ of (20b) for population imbalance $\sigma _i$ reads as [cf. (4)]

21 $$\begin{aligned} \begin{aligned} \sigma _i\simeq \sigma _{0,i}\frac{ \gamma _{p,i}+\gamma _{e,i}}{\gamma _{p,i}+\gamma _{e,i}+4\kappa C_i|E_i|^2}. \end{aligned} \end{aligned}$$

Linearizing (21) and substituting it into (20a), we obtain

22 $$\begin{aligned} \begin{aligned} \dot{E}_i= -i\Delta _i E_i + \kappa (C_i\sigma _{0,i}-1) E_i + iJ\sum _i{A_{ij}E_j} - \frac{4 \sigma _{0,i} \kappa ^2 C_i^2 |E_i|^2 E_i}{\gamma _{p,i}+\gamma _{e,i}}, \end{aligned} \end{aligned}$$

where $C_i$ is defined in (8).

Equation (20) and their simplified version Eq. (22) describe the diffusion of information in the DIS, cf.^27^. As seen from Eq. (22), the enhancement of socially actual information in the DIS essentially depends on generalized NIAs cooperativity parameter $G_i$ that accounts for population imbalance $\sigma _{0,i}$ indicating the inherent preferences of NIAs without coupling with AIAs. The social lasing (information enhancement) effect occurs at

23 $$\begin{aligned} \begin{aligned} G_i\equiv \sigma _{0,i} C_{i}>1. \end{aligned} \end{aligned}$$

**Figure 7:**
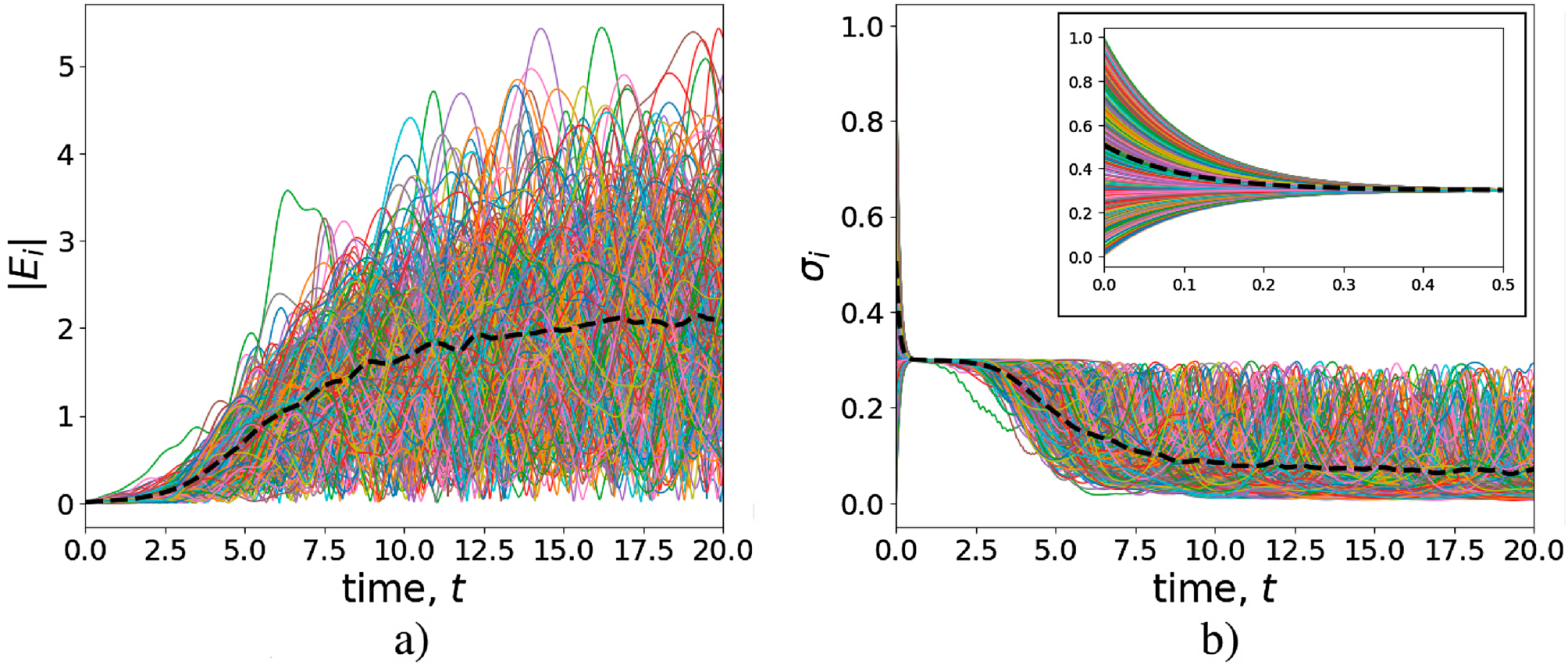
Dependence of (**a**) s-field amplitude absolute value $|E_i|$ and (**b**) population imbalance $\sigma _i$, $i=1,\ldots ,N$ vs. dimensionless time variable *t*. The black dashed curves correspond to average values $|\bar{E}|$ and $\bar{\sigma }$, respectively. The parameters are $N = 300$, $g_i = g = 1$, $\kappa = 0.1$, $(\gamma _p+\gamma _e) = 10$, $\sigma _{0,i} = 0.3$; $\Delta _i\in [-0.1;0.1]$ and $\Gamma _i\in [0.2;0.4]$ are random and uniformly distributed variables. The initial condition for $E_i$ at $t=0$ we took as $E_i(t=0) = 0.1$; $\sigma _i(t=0)$ is randomly and uniformly distributed within $\sigma _i(t=0) \in [0;1]$. Algorithm S2 in Supplementary information explains the details.

In Fig. 7, we represent the numerical solutions of Eq. (20), which reflect the particular choices of $N=300$ avatars and their users. In particular, avatar preferences result in the s-field enhancement as seen in Fig. 7. The dashed (solid black) curve in Fig. 7a establishes the dependence for average s-field absolute value $|\bar{E}|$, where $\bar{E}$ defined [cf. (13)]

24 $$\begin{aligned} \begin{aligned} \bar{E}=\frac{1}{N\langle k\rangle }\sum _{i=1}^{N} k_i E_i. \end{aligned} \end{aligned}$$

Figure 7b demonstrates the relevant behavior of average population imbalance $\sigma _i$. Average value $\bar{\sigma }$ defined in (3) is shown as the dashed (solid black) curve and characterizes the average social polarization of the NIAs community in the DIS. We take the initial values for $\sigma _i$ uniformly distributed in the domain, $0\le \sigma _i\le 1$, we are interested in this work. Noteworthy, the transition to the s-field enhancement is independent on the initial users’ opinion variety: the initial distribution of $\sigma _i$ rapidly collapses to value $\sigma _i\simeq \sigma _{0,i}$, see the inset in Fig. 7b. Then, one can recognize the results previously obtained within time domain $0.4\le t \le 0.9$ where the s-field and population imbalance do not change in time. Starting from $t\simeq 1$, s-field amplitudes $E_j$ grow, while $\sigma _i$ vanish. Fig. 7a exhibits oscillations within this limit. $\bar{E}$ approaches an average level of discussions that occur in the DIS at large *t*. As follows from Fig. 7a, the establishment of average s-field (opinion formation) occurs due to the high level of communication and cooperation; the generalized cooperativity parameters, $G_i$, for the curves in Fig. 7 obey condition (23). Simultaneously, the average social polarization, $\bar{\sigma }$, tends to zero, which indicates some small resistant social polarization that occurs in the DIS at large *t*. Noteworthy, the users’ states in this case can be recognized as some relaxed ones. Noteworthy, this, socially preferable, limit has been studied in detail in the framework of cooperation emergency in evolutionary games^38^. In our case, one can speak about the long-term adaptivity of AIAs to the NIAs community due to the occurrence of non-vanishing average s-field shown in Fig. 7a.

**Figure 8:**
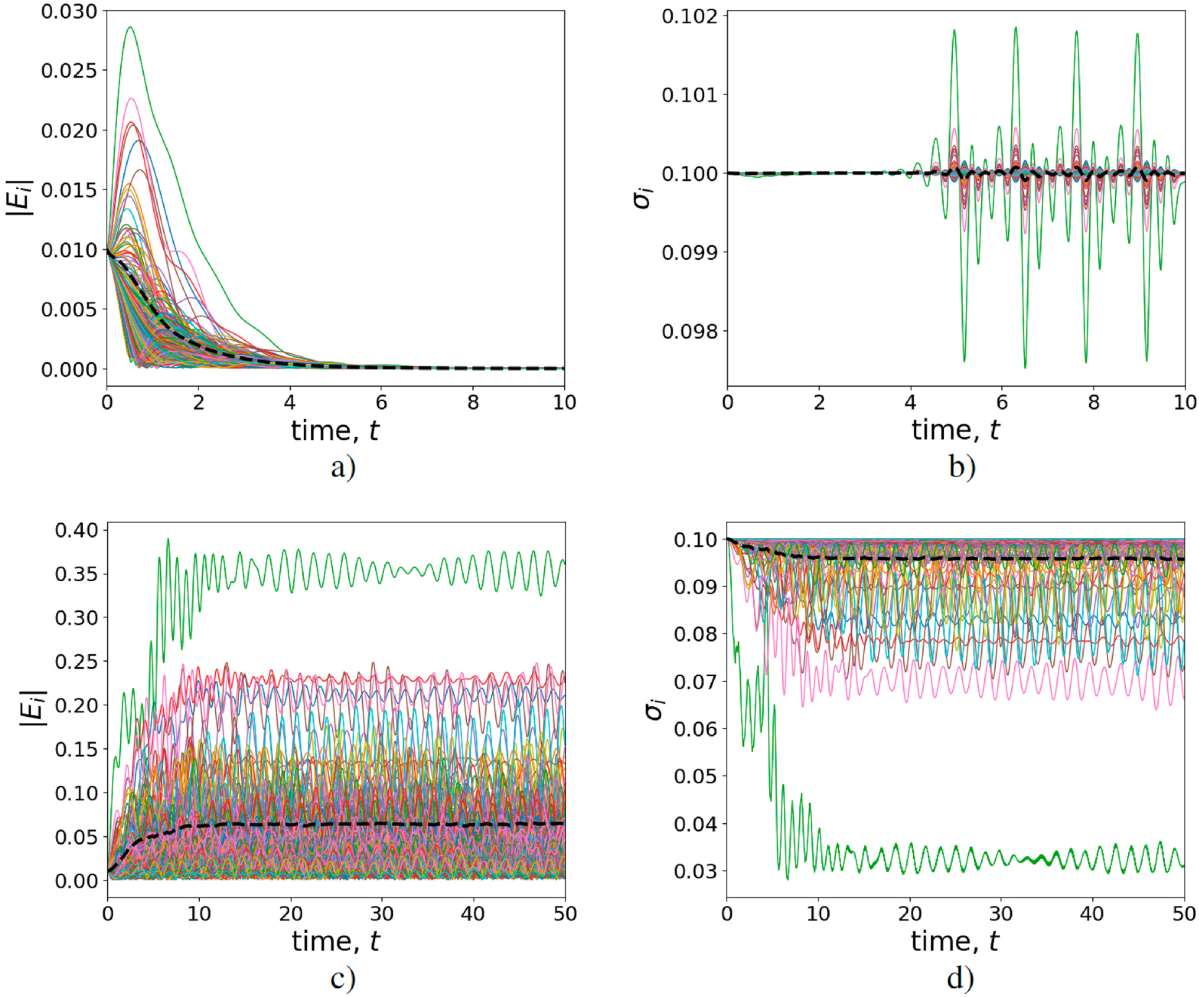
Dependence of (**a**,**c**) s-field amplitude absolute value $|E_i|$ and (**b**,**d**) population imbalance $\sigma _i$ vs. dimensionless time *t*. The black dashed curves correspond to average values $|\bar{E}|$ and $\bar{\sigma }$, respectively. For (**a**,**b**) $g_i = g = 1$; for (**c**,**d**) $g_i = \sqrt{k_i}$. The other parameters are $N = 300$, $g=1$, $\kappa =1$, $\gamma _p+\gamma _e = 10$; $\Gamma _i\in [0.2;1.8]$ and $\Delta _i\in [-0.1;0.1]$ are random and uniformly distributed variables. The initial conditions are $E_i(0) = 0.01$, $\sigma _i(0) = \sigma _{0,i} = 0.1$.

However, in the limit of small cooperativity parameters $C_i$ no aforementioned long-term adaptivity of AIAs occurs. Fig. 8 illustrates the features of opinion formation in the DIS close to the phase transition point defined by condition (17). In particular, Fig. 8a determines the opinion formation above the threshold and is relevant to the parameters taken at $\sigma _i(0) = \sigma _{0,i} = 0.1$ for the main sketch in Fig. 5. The features of the curves in Fig. 8 demonstrate the initial growth of the s-field as it occurs in Fig. 7a. However, then the s-field vanishes approaching value $|\bar{E}|=0$. Such a behavior may be explained by the inhomogeneity of generalized cooperativity parameter $G_i$ for different users and avatars in the DIS. For some nodes, $G_i>1$ that implies the s-field enhancement. Although at large time slots, the uncertainties coming from small amplitude persistent oscillations and revivals of $\sigma _{0,i}$ (see the inset in Fig. 8a finally make it impossible to support the finite (non-zero) information field in the DIS.

Noteworthy, network-dependent AIA–NIA coupling rate $g_i=\sqrt{k_i}$ significantly modifies this picture, see Fig. 8b,c. An improvement of the cooperativity parameters for a large number of avatars leads to overcoming phase transition level (17) inherent to the DIS; the s-field exhibits inhomogeneous amplification, see Fig. 8b,c and cf. Fig. 7. The green curves in Fig. 8 correspond to the hub that possesses maximal $k_i$, see the highest blue dots in Fig. 3. Thus, the hierarchy of avatars and users’ features appears as a result of their position in the DIS network.

## Methods

### DIS network model

**Figure 9:**
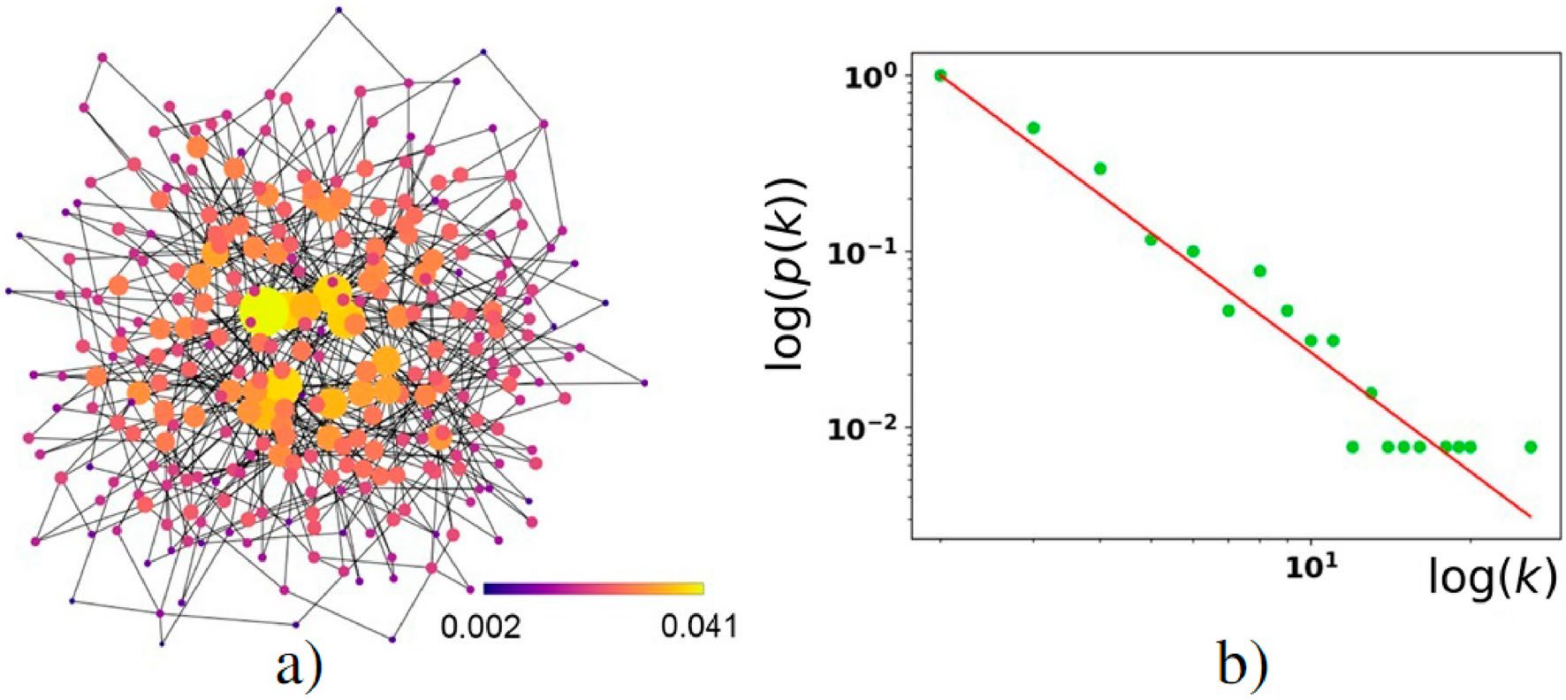
(**a**) Avatar–avatar network, (**b**) node degree distribution in a double logarithm scale that demonstrates power-law-like distribution (25) with $\eta = 2.255$; $k_{min} = 2$; $k_{max} = 26$; $\langle k\rangle \simeq 4$, $\zeta \simeq 6.57$, $N=300$. Algorithm S3 in Supplementary information explains the details.

In Fig. 9a, the avatar–avatar network is represented as an undirected graph. We assume the network provides *N* nodes and satisfies a power-law degree distribution

25 $$\begin{aligned} \begin{aligned} p(k)=\frac{(\eta -1)k_{min}^{\eta -1}}{k^{\eta }}, \end{aligned} \end{aligned}$$

that obeys the normalization condition

26 $$\begin{aligned} \int \limits ^{+\infty }_{k_{min}}{p(k)dk}=1. \end{aligned}$$

In (25) $\eta$ is the degree exponent that determines the topology of the network; $k_{min}$ is the smallest degree. The properties of networks possessing distribution (25) for $\eta =2$ and $\eta =3$ should be calculated separately,^41^. Noteworthy, practically meaningful networks admit scale-free domain $2\le \eta \le 3$ for $\eta$ parameter, where hubs occur, e.g.^65^. The largest hub for the network described by distribution (25) is determined by maximal node degree $k_{max}=k_{min}e^{\frac{1}{\eta -1}}$ and called a natural cutoff.

We represent *p*(*k*) in Fig. 9b. The hubs in Fig. 9b are characterized by the straight group of green dots, which are displayed at the bottom of the right corner of the distribution function. In this work we suppose that degree exponent $\eta$, *N*, $k_{min}$ and $k_{max}$ are time-independent. This approach looks reasonable for the time slots when the number of users in DIS represents some close (target) community and is constant on average. In other words, we examine the DIS properties for a given feature of avatar–avatar network.

For Fig. 9 we used the **igraph** library of **R** to generate the graph; **NetworkX** of the Python library to work with the graph; **Matplotlib** for the graph visualization; and **Numpy** of the Python library to find eigenvalues and eigenvectors. The combination of **R** and Python leverages the statistical rigor and versatility provided by both programming environments. The **R**’s **igraph** package offers efficient data handling and preliminary analysis capabilities, while Python’s **NetworkX** allows for extensive simulations and dynamic analyses, crucial for modeling the interactive dynamics of the DIS. The **Matplotlib** supports these analyses with high-quality visualizations, facilitating detailed examination, and presentation of results. This methodological framework ensures the comprehensive exploration and robust validation of the network’s structural and dynamic properties.

To validate the scale-free nature of the network in Fig. 9, we assess the degree distribution for adherence to a power law using goodness-of-fit tests. A close fit confirms the network structural validity under the Barabási-Albert model. The stability and consistency of the eigenvector centrality scores across various network configurations and parameter settings are evaluated to ensure reliability in identifying influential nodes.

The information about the graph in Fig. 9a is stored in symmetric adjacency matrix $A_{ij}$. In particular, matrix element $A_{ij}=1$ if two *i* and *j* nodes are linked and $A_{ij}=0$ otherwise. We characterize statistical properties of the network by the first ($\langle k\rangle$) and normalized second ($\zeta$) moments of the node degree defined as

27a $$\begin{aligned} \langle k\rangle&=\frac{1}{N}\sum _{i=1}^{N} k_i= \frac{1}{N}\sum _{i,j}^{N} A_{ij}; \end{aligned}$$

27b $$\begin{aligned} \zeta&\equiv \frac{1}{N\langle k\rangle }\sum _i{k_i^2}, \end{aligned}$$

where $k_{i}$ is the *i*-th node degree.

In this work, we use the eigenvector centrality criterion that we can represent in the form (e.g.^62^)

28 $$\begin{aligned} \lambda _{p} \mathscr {E}_{p,i}= \sum _{j=1}^{N}{A_{ij}\mathscr {E}_{p,j}}, \end{aligned}$$

where $\lambda _{p}$ is the PE; $\mathscr {E}_{p,i}$ ($i=1,\ldots ,N$) is the correspondent eigenvector components. The Perron–Frobenius theorem states real $\mathscr {E}_{p,i}$. The value of each *i*-th component specifies the color/size of the *i*-th node in Fig. 9a.

### Quantum-inspired model of DM agents

In the framework of the considered DIS model, we establish the mental state of each NIA by two mutually orthogonal states in Hilbert space, ground (relaxed) $|g\rangle _i$ and excited $|e\rangle _i$, see Fig. 2a; these states obey conditions

29 $$\begin{aligned} {_j}{\left\langle g |g\right\rangle }{_i}={_j}{\left\langle e |e\right\rangle }{_j}=\delta _{ij}, \quad {_j}{\left\langle g |e\right\rangle }{_i}=0. \end{aligned}$$

In the framework of the quantum field approach, it is possible to introduce number operators $\hat{n}_{e,i}\equiv |e\rangle _{ii}\langle e|$ and $\hat{n}_{g,i}\equiv |g\rangle _{ii}\langle g|$ for the *i*-th user that we can recognize as DM operators, cf.^12^. In particular, they are relevant to $\mathscr {S}_e$ and $\mathscr {S}_g$ decisions, respectively, and possess 0 and $+1$ eigenvalues, as it follows from (29);

30 $$\begin{aligned} \hat{n}_{g,i}|g \rangle _i= +1 |g \rangle _i, \quad \hat{n}_{g,i}|e \rangle _i=0, \quad \hat{n}_{e,i}|e\rangle _i=+1 |e \rangle _i, \quad \hat{n}_{e,i}|g \rangle _i=0. \end{aligned}$$

We use Eq. (30) in the mean-field approach applied throughout the work.

Taking into account Eqs. (29), (30), we can determine relatively simple rules followed by NIAs in the DIS when interacting with AIAs. These rules are quite similar to those that we formulated in^27^ in the framework of the solaser approach.

First, let us examine the *i*-th NIA’s basic features in emotionally relaxed (ground) state $|g\rangle _i$. We propose that the *i*-th user becomes excited in two primary cases. In particular, a user might obtain some (emotionally excited) information from the outside of the network with rate $\gamma _{p,i}$, which can excite their emotional state, Fig. 2. In other words, $\gamma _{p,i}$ represents an analog of the classical pump field that helps create population inversion in the usual laser medium, see e.g.^59^.

Another way of NIA’s mental excitation is connected with the interaction of the *i*-th user with their avatar that presumes to obtain some recommendations. These recommendations are realized using short messages, which carry some social energy $\hbar \Omega _{i}$, $i=1,\ldots ,N$. Following the social laser approach, we propose that any meaningful message forms a quantum of socially significant information (s-photon), which may be absorbed by the user in the ground state $|g\rangle _i$, cf.^27^. The s-photons are thematically “colored” through frequencies $\Omega _i$. We suppose that there exists a probability to change the user’s emotional state to the excited one, $|e\rangle _i$, if they approve the message obtained from the avatar and support the $\mathscr {S}_e$ opinion with a relevant probability. In this case, we can assume that the user absorbing an s-photon accumulates some additional (positive) emotions relevant to some social energy in our interpretation, see Fig. 2b. On the other hand, the users stay in emotionally relaxed (ground) states $|g\rangle _i$ if they dislike $\mathscr {S}_e$ or are indifferent to the message served by their avatars. The message is also considered unpleasant if it is irrelevant to the user. In this case, the user ignores or loses the message. This assumption looks reasonable since NIAs do not change their ground state in this case.

Second, we assume that when in an emotionally excited state $|e\rangle _i$, the *i*-th NIA is guided by the following simple rules of behavior. The *i*-th user can relax and transfer their emotional state to the ground one, $|g\rangle _i$. Thus, the quantum-like description of NIA’s cognitive abilities in the framework of the solaser paradigm presumes the existence of some characteristic time $\tau _{e,i}$ that NIAs (humans) can spend in the emotionally excited state. In the paper, we use the inverse parameter, $\gamma _{e,i}\simeq 1/\tau _{e,i}$, that characterizes the rate of the excited state $|e\rangle _i$ decay.

The user can transfer to emotional ground state $|g\rangle _i$ changing their excited state $|e\rangle _i$ in two other cases. First, a user can “spontaneously” emit an s-photon (send some message to their avatar, for example). Second, a user obtaining a message from the avatar immediately emits s-photon changing the state to the $|g\rangle _i$ one. All these facilities of NIAs reminiscent properties of two-level atoms in the presence of quantized irradiation^52^. In this sense, we can recognize NIAs as so-called social atoms of the DIS^51^.

### Mean-field equations

Hamiltonian (1) leads to Heisenberg–Langevin operator equations. In this work, we are interested in the mean-field approximation that excludes quantum field fluctuations and relevant correlations with NIAs. After straightforward calculations, which account (29), (30), for (1), we obtain set of equations

31a $$\begin{aligned} \dot{\alpha }_i&=(-i\Omega _i-\kappa )\alpha _i-ig_i p_i+iJ\sum _{j=1}^{N}{A_{ij}\alpha _j}; \end{aligned}$$

31b $$\begin{aligned} \dot{p}_i&=(-i\Omega _{0,i}-\Gamma _i)p_i+ig_i\sigma _i\alpha _i; \end{aligned}$$

31c $$\begin{aligned} \dot{\sigma }_i&= (\gamma _{p,i}-\gamma _{e,i})-(\gamma _{p,i}+\gamma _{e,i})\sigma _i+2ig_i(\alpha _i^*p_i-\alpha _ip_i^*), \end{aligned}$$

where $\alpha _i=\langle \hat{a}_i\rangle$, $p_i=\langle \hat{\sigma }^-_i \rangle$, and $\sigma _i =\langle \hat{\sigma }_i^z \rangle$ are the mean-field variables, $i=1,2, \ldots , N$; $\Gamma _i$ is the *i*-th user excitation decay rate; $\kappa$ characterizes information losses in the DIS. For our purposes, it is fruitful to remove rapidly oscillating components in Eq. (31): substituting $p_i(t) = P_i e^{-i\Omega _{0,i} t},\, \alpha _i(t) = E_i e^{-i\Omega _{0,i} t}$ into Eq. (31), we immediately obtain Eq. (2).

In this work, we are interested in stationary solutions $P_i(t) = \mathscr {P}_i e^{-i\omega t},\, E_i(t) = \mathscr {E}_i e^{ - i\omega t}$ of Eq. (31) assuming $\sigma _{i}\simeq \sigma _{0,i}$, see (4). Substituting $P_i(t)$ and $E_i(t)$ into (31), we obtain

32a $$\begin{aligned}&(\omega - \Delta _i + i\kappa )\mathscr {E}_i - g_i\mathscr {P}_i + J\sum _{j=1}^{N}{A_{ij}\mathscr {E}_j} = 0; \end{aligned}$$

32b $$\begin{aligned}&(i\Gamma _i + \omega )\mathscr {P}_i + g_i\sigma _{0,i} \mathscr {E}_i = 0. \end{aligned}$$

Resolving Eq. (32b) for excitation $\mathscr {P}_i$ and substituting it into (32a), we immediately obtain (5).

### Discussion

Let us summarize the results obtained. In this work, we have suggested the quantum-inspired model of DIS that consists of two types of agents that are NIAs (users) and their digital assistants (avatars) AIAs, see Fig. 1. The avatars can exchange information in the avatar–avatar network that possesses *N* nodes; we recognize this network as an undirected graph described by the power-law degree distribution and possessing hubs, see Fig. 9. We have mapped Russell’s circumplex model of valence and arousal to an effective two-level quantum system to specify the NIAs’ cognitive abilities in the framework of a simple (binary) decision-making process, cf. Fig. 2. The users can obtain information from the outside; their cognitive states and preferences are characterized by the mean value of inversion operator $\sigma _i$, which describes an average mental (emotional) state of the *i*-th user to the information represented by their avatar. The domain we have considered in this work, $0\le \sigma _i\le 1$, indicates the role of an external information pump that can excite individuals in the DM process. The allowed frequencies characterize the variety of opinions in the DIS; the opinion gap occurs as a result of the avatar–user interaction in each node of the network and manifests two-branch opinions. Practically, the opinion gap indicates shifting the user’s emotional state, opinions, and beliefs. Some of them may be reinforced or, on the contrary, rapidly vanish. In this work, taking into account avatar–avatar coupling within the network, we have proposed clear criteria for how it happens.

We have demonstrated that a nonvanishing macroscopic coherent information field in the DIS occurs in the case when the users are satisfied by their avatars. In particular, the opinion formation and social impact in the DIS are relevant to the second-order (laser-like) phase transition similar to the social laser phenomenon in social networks, cf.^27^. Otherwise, the rapidly decaying information field manifests the absence of any collective decisions for the avatars in the DIS. Long-term adaptivity of avatars to their users cannot be implemented in this limit.

We have shown that a set of cooperativity parameters $C_i$, $i=1,\ldots , N$ for the users in DIS plays a significant role in the DIS features and establishes the necessary condition to achieve the discussed phase transition. The cooperation between users is realized through their digital assistants. Practically, each cooperativity parameter depends on the coupling of a particular NIA with relevant AIA manifesting how frequent their communication.

A sufficient condition for the opinion formation and social impact is characterized by the generalized cooperativity parameter $G_i$ that also accounts for the user’s individual preferences and external information, embodied in $\sigma _i$. We have shown that the phase transition to opinion formation and social impact in the DIS occurs if a large number of avatar–user pairs fulfill the lasing-like transition criterion (17). In this case, the opinions relevant to the $\texttt {Im}(\omega )>0$ condition are enhanced in the DIS; other opinions with $\texttt {Im}(\omega )<0$ are suppressed, see Fig. 6. Dynamically, in this limit, the socially actual s-filed is amplified and information diffusion occurs in the DIS, e.g. Fig. 7. Noteworthy, the avatars that fall into domain $-g< \texttt {Re}(\omega ) < g$ tightly cooperate with their users and tend to focus on suggestions supported by information pumping with increasing $\sigma _i$. Noteworthy, such avatars possess small $k_i$ within the avatar–avatar network.

Noteworthy, the avatar–avatar network peculiarities play a remarkable role in the opinion formation in the DIS. We have shown that in complex networks, avatars tend to self-organize, which accounts topological properties of the network in a scale-free regime. In this limit, coupling between some of the avatars effectively becomes much stronger than with their users. Such avatars in the avatar–avatar network correspond to maximally influential AIAs, which tend to maintain their influence in the DIS.

For weak AIA–NIA coupling strength ($C_i\le 1$) there are many uncertainties in the DIS, which makes it difficult for the DM agents to form a certain (non-zero) opinion, cf. Fig. 5. Below the phase transition threshold for the DIS parameters, the s-field vanishes, which indicates the inhibition of avatars adaptation to their users. We have shown that the avatar–avatar network peculiarities play a remarkable role in this limit. In particular, we have demonstrated that in complex networks, avatars tend to self-organize, which accounts topological properties of the network in a scale-free regime. In this limit, coupling between some of the avatars effectively becomes much stronger than with their users. Such avatars in the avatar–avatar network correspond to maximally influential AIAs, which tend to maintain their influence in the DIS.

We have suggested a procedure that adaptively increases the users’ impact by establishing a network-dependent coupling rate with their digital assistants in the limit of weak AIA–NIA coupling strength. In this case, the *i*-th cooperativity parameter, $C_i$, may be significantly improved in $k_i$ times, see Fig. 3. In particular, the suggested mechanism of AIA adaptive control helps resolve the DM process in the presence of some uncertainties evoked by the users’ preferences, cf. Fig. 6c,d. Thus, our approach generally provides a clear way to control AIAs, distributed within complex network systems.

Our findings open new prospects in different areas where AIAs can potentially represent useful teammates for humans to solve common routine problems in the framework of some network organizations. As mentioned above, some specific networks could be interesting in economy and finance (marketplaces, stocks), professional and business activities (office networks), etc. In this regard, the DIS we have examined in this work should be also considered in the framework of evolutionary game theory when the cooperative behavior of DM agents represents a primary interest, see e.g.^35,42^. We suppose that the cooperativity parameters introduced in this work may be adopted for the evolutionary game within some limits in our problem. Noteworthy, quantum probability formalism can also be useful here to characterize the non-rational behavior of DM agents, cf.^66,67^. This problem requires a separate analysis.

Notably, arbitrary networks require accounting for the primary emotional (cognitive) states of DM agents that the Russell circumplex model represents. In other words, a simple two-level model (see Fig. 2) should be revised; we can speak about four- or three-level models as possible generalizations of the two-level (mental) model we consider in this work. Additional levels are necessary to specify cognitive (emotional) states that possess negative valence in Fig. 2a. In quantum physics, three- and four-level systems are typically used for the elucidation of coherent features of atomic systems that simultaneously interact with two or more fields, e.g.^52^. In our case, three- and four-level models can describe cognitive states of NIA inherent to all quarters of the Russell circumplex model. Notice, that more accurate (multilevel) models may be also designed for other approaches to human basic emotional states (specified by Ekman and Plutchik). Thus, the generalization of the two-level model multilevel cognitive system for mental state specification represents a challenging task for us to investigate.

### Supplementary Information

Supplementary Information.

## Data Availability

All data generated or analysed during this study are included in this published article and Supplementary Information.
