# Supplementary material for: Quantum-inspired modeling of distributed intelligence systems with artificial intelligent agents self-organization
Source: Sci Rep. 2024 Jul 4;14:15438. doi: 10.1038/s41598-024-65684-z (PMC11224413; doi:10.1038/s41598-024-65684-z)
Supplement: Supplementary file 1 — Supplementary Information. [file 41598_2024_65684_MOESM1_ESM.pdf]

Supplementary information for "Quantum-inspired modeling of distributed intelligence systems with artificial intelligent agents self-organization"

Algorithm S1 Computation of dimensionless imaginary parts `Im`(ω) vs. `Re`(ω) of the set of $2N$ eigenfrequencies ω for data represented in Fig.4b

$N \leftarrow 300$ ▷ Number of nodes
$\eta \leftarrow 2.255$ ▷ Power-law exponent for the network degree distribution
$G \leftarrow$ nx.barabasi_albert_graph(N, η)
$A \leftarrow$ nx.adjacency_matrix$(G).toarray()$
$k \leftarrow A.sum(\text{axis} = 1)$ ▷ Degree of each node
$\Delta \leftarrow$ np.random.uniform$(-0.1, 0.1, N)$ ▷ Random Δ values
$\Gamma \leftarrow$ np.random.uniform$(0.2, 0.4, N)$ ▷ Random Γ values
$\sigma_{0,i} \leftarrow 0.3$ ▷ Constant value for all nodes
$\kappa \leftarrow 0.1 \times$ **ones**(N) ▷ Array of decay rates
$J_{values} \leftarrow [0, 0.5, 2]$ ▷ List of J values
Initialize $Matrixcoef$ as a dictionary of zero matrices for each J
for J in J_{values} **do**
    for i in 1 to N **do**
        for j in 1 to N **do**
            $Matrixcoef[J][i, j] \leftarrow 1j \times J \times A[i, j]$
        end for
        $Matrixcoef[J][i, i] \leftarrow -1j \times \Delta[i] - \kappa$
    end for
    for i in $N + 1$ to $2N$ **do**
        $Matrixcoef[J][i, i - N] \leftarrow 1j \times \sigma_{0_z} \times k[i - N]$
        $Matrixcoef[J][i, i] \leftarrow -\Gamma[i - N]$
    end for
end for
$eigenvalues \leftarrow \{\}$
for J in J_{values} **do**
    $eigenvalues[J] \leftarrow$ np.linalg.eig$(Matrixcoef[J])[0]$
end for
Plot results

Algorithm S2 Computation of s-field amplitude absolute value $|E_i|$ and population imbalance σ_i vs. dimensionless time t for data represented in Fig. 7.

Initialize parameters:
$N \leftarrow 300$ ▷ Number of nodes
$g \leftarrow 1.0$ ▷ Coupling rate
$J \leftarrow 0.5$ ▷ Interaction strength
$\gamma_p + \gamma_D \leftarrow 10.0$ ▷ Sum of pumping and decay rates
$\sigma_{0,i} \leftarrow 0.3$ ▷ Baseline inversion
$\kappa \leftarrow 0.1 \times \mathbf{ones}(N)$ ▷ Array of decay rates
$\Delta \leftarrow \mathbf{random.uniform}(-0.1, 0.1, N)$ ▷ Detuning array
$\Gamma \leftarrow \mathbf{random.uniform}(0.2, 0.4, N)$ ▷ Damping array
Define system dynamics:
function SYSTEM(t, Y)
    $E \leftarrow Y[: N]$
    $\sigma_z \leftarrow Y[N :]$
    $k \leftarrow \mathbf{sum}(A, \text{axis} = 1)$
    $sumE \leftarrow \mathbf{dot}(tau, E)$
    $dE\_dt \leftarrow (-1j \cdot \Delta - \kappa) \cdot E + \frac{\sigma_z \cdot E}{\Gamma} + 1j \cdot J \cdot sumE$
    $dsigma\_dt \leftarrow (\sigma_0 - sigma_z) \cdot \gamma_p \cdot \gamma_D - 4 \cdot \frac{sigma_z \cdot |E|^2}{\Gamma}$
    return concatenate$(dE\_dt, dsigma\_dt)$
end function
Initialize simulation:
$E_0 \leftarrow (0.01 + 0j) \times \mathbf{ones}(N)$
$sigma\_z0 \leftarrow \mathbf{random.uniform}(0, 1, N)$
$Y0 \leftarrow \mathbf{concatenate}(\mathbf{full}(N, E0), \mathbf{full}(N, sigma\_z0))$
$t\_span \leftarrow (0, 20)$
$sol \leftarrow \mathbf{solve\_ivp}(system, t\_span, Y0, t\_eval = \mathbf{linspace}(t\_span[0], t\_span[1], 1000), \text{method} =' RK45')$
$t \leftarrow sol.t$
$E \leftarrow sol.y[: N]$
$\sigma \leftarrow sol.y[N :]$
Plot results

Algorithm S3 Generation of avatar – avatar network graph and analysis of its node degree distribution for data represented in Fig. 9.

Generate Graph:
$n \leftarrow 300$ ▷ Number of nodes
$m \leftarrow 2$ ▷ Number of edges to attach from a new node to existing nodes
$\eta \leftarrow 2.255$ ▷ Power-law exponent for the network degree distribution
$G \leftarrow$ barabasi_game($n, power = \eta, m = m, directed = False$)
Plot the graph G with a circular layout
Calculate Degree Distribution:
$degree\_dist \leftarrow$ degree(G)
$power\_fit \leftarrow$ power.law.fit($degree\_dist$)
Eigenvector Centrality Calculation:
$A \leftarrow$ adjacency_matrix(G) ▷ Get the adjacency matrix of G
$eigenvalues, eigenvectors \leftarrow$ compute_eigenvalues_and_vectors(A)
$max\_index \leftarrow$ index_of_maximum_eigenvalue($eigenvalues$)
$max\_eigenvector \leftarrow$ absolute_values($eigenvectors[:, max\_index]$)
Display eigenvector centrality using $max\_eigenvector$
Node Degree Distribution:
Prepare lists x and y for plotting
$degree_{c}ounts \leftarrow$ frequency_count($degree\_dist$)
for $degree$ in sort_keys($degree\_counts$) **do**
    Add $degree$ to x
    Add $degree\_counts[degree]/len(G)$ to y
end for
Plot the degree distribution on a log-log scale
Plot Approximation Line:
Calculate $line\_y$ for the approximation line:
$line\_y \leftarrow 2 \cdot m^{(\eta-1)} \cdot x^{-\eta}$
Plot $x, line\_y$ on the same graph as the degree distribution
